# Ndrg3 is a critical regulator of peripheral T cell maturation and homeostasis

**DOI:** 10.1126/sciadv.ads5143

**Published:** 2025-03-12

**Authors:** Julia A. Komorowska, Christiane Grammer, Mirela Bălan, Jeremy B. Swann

**Affiliations:** ^1^ Department of Developmental Immunology, Max Planck Institute of Immunobiology and Epigenetics, Freiburg, Germany.; ^2^ Faculty of Biology, Albert Ludwig University, Freiburg, Germany.; ^3^ Bioinformatics Core Facility, Max Planck Institute of Immunobiology and Epigenetics, Freiburg, Germany.

## Abstract

To provide protection, anticipatory T cell–dependent immunity is reliant on the generation and maintenance of a naïve T cell repertoire, which is sufficiently diverse to ensure recognition of newly encountered antigens. Therefore, under steady-state conditions, a given individual needs to maintain a large pool of naïve T cells, ready to respond to potential threats. Here, we demonstrate that N-myc downstream-regulated gene 3 (Ndrg3) is essential for naïve T cell stability. Mice with T cell–specific Ndrg3 loss are lymphopenic, with reduced numbers of conventional T cells and natural killer T cells. We show that in the absence of Ndrg3, naïve CD8+ T cells exhibit high rates of both proliferation and apoptosis, phenotypes that could be partially rescued by transgenic expression of a high-avidity T cell receptor. Furthermore, Ndrg3-deficient cells were refractory to interleukin-4, resulting in reduced Eomes induction, and a decreased virtual memory population. Our study therefore identifies Ndrg3 as an unexpected, pleiotropic regulator of T cell homeostasis.

## Teaser

Ndrg3 maintains naïve T cells in a quiescent and viable state in secondary lymphoid organs.

## INTRODUCTION

The peripheral T cell pool is a heterogeneous population, made up of cells in varying states of differentiation, which exhibit a range of phenotypic and functional characteristics (*1*, *2*). The diversity of the T cell populations is dictated by maturation and maintenance processes that occur both within the thymus, which generates cells with new specificities (*3*), and in the periphery where established cells are maintained and respond to potential threats (*4*). Although many fundamental features of T cell development, such as T cell receptor (TCR) rearrangement (*5*) and commitment to CD4 and CD8 subsets (*6*), are fixed during development within the thymus, other attributes and functional specializations are acquired during postthymic maturation (*7*–*9*), which occurs in secondary lymphoid organs (*10*) and is crucial for establishing and maintaining stable peripheral T cell homeostasis (*11*).

The initial establishment of the peripheral T cell pool occurs early in development, and at this time, the bulk of cells emigrating from the thymus [referred to as recent thymic emigrants (RTEs)] is incorporated into the incipient T cell repertoire (*12*). Once the peripheral T cell niche is filled, homeostatic numbers of T cells need to be maintained, and this process involves both ongoing, low-level recruitment of RTEs, as well as the long-term preservation of existing naïve and memory cells. The active maintenance of naïve T cell quiescence requires the strict control of survival and mitogenic signals to avoid inducing cell differentiation. Interleukin-7 (IL-7) (*13*) and tonic TCR signaling (*14*, *15*) plays central roles in ensuring the survival and slow-paced turnover of naïve cells. In addition to IL-7 and tonic signaling, several other pathways and factors help to ensure the stability of naïve T cells. A number of transcriptional modulators are required to ensure appropriate gene expression by naïve T cells, and transcriptional repressors [nuclear factor κB–activating protein (*16*)], histone modifiers [histone deacetylase 3 (*17*)], transcription factors [runt-related transcription factor 1 (*18*), zinc finger protein 335 (*19*), and forkhead box protein O1 (*20*)], and transcriptional co-regulators [Ess2 (*21*)] are all necessary to establish and maintain the naïve T cell pool. The survival of naïve T cells in the periphery also requires the activity of the apoptotic regulators IκB kinase 2 and receptor-interacting protein kinase 1 (*22*–*25*), and control of cellular metabolism is key to acquiring a quiescent, mature naïve state (*26*–*28*).

Here, we identify Ndrg3 as a previously unknown regulator of postthymic T cell maturation. The N-myc downstream-regulated gene family (Ndrg) includes four members, Ndrg1 to Ndrg4, which are characterized by a defining Ndr domain (*29*). This Ndr domain forms an αβ-hydrolase fold-like structure; however, these proteins are thought to be nonenzymatic, as they lack the catalytic triad required for hydrolytic activity (*30*–*32*). Ndrg-family members have been implicated in a number of cellular and developmental processes, and accumulating evidence demonstrates that these proteins have important roles in T cell biology. Experiments in zebrafish have shown that ndrg1b expression can rescue T cell development in phosphatase and tensin homolog (PTEN)–deficient fish (*33*), and in mice and humans, Ndrg1 expression is up-regulated during anergy induction, although there are differing interpretations as to the functional role of Ndrg1 under these conditions (*34*, *35*). Furthermore, Ndrg2 has been identified as a tumor suppressor in adult T cell leukemia/lymphoma (*36*), where it is thought to modulate phosphatidylinositol 3-kinase (PI3K)-Akt signaling. Our interest in Ndrg3 was raised by the early observation that this gene was highly expressed in the embryonic thymus (*37*), and transcriptional profiling of immune cells has revealed that relative to other Ndrg-family members, Ndrg3 is highly expressed in T cells (*38*). To extend upon these observations, we have examined the consequences of Ndrg3 deficiency in T cells and found that Ndrg3 plays a critical role in promoting the survival and quiescence of naïve T cells in peripheral lymphoid organs.

## RESULTS

### Phenotypically normal thymocyte development in Ndrg3-deficient thymi

**Fig. 1.**
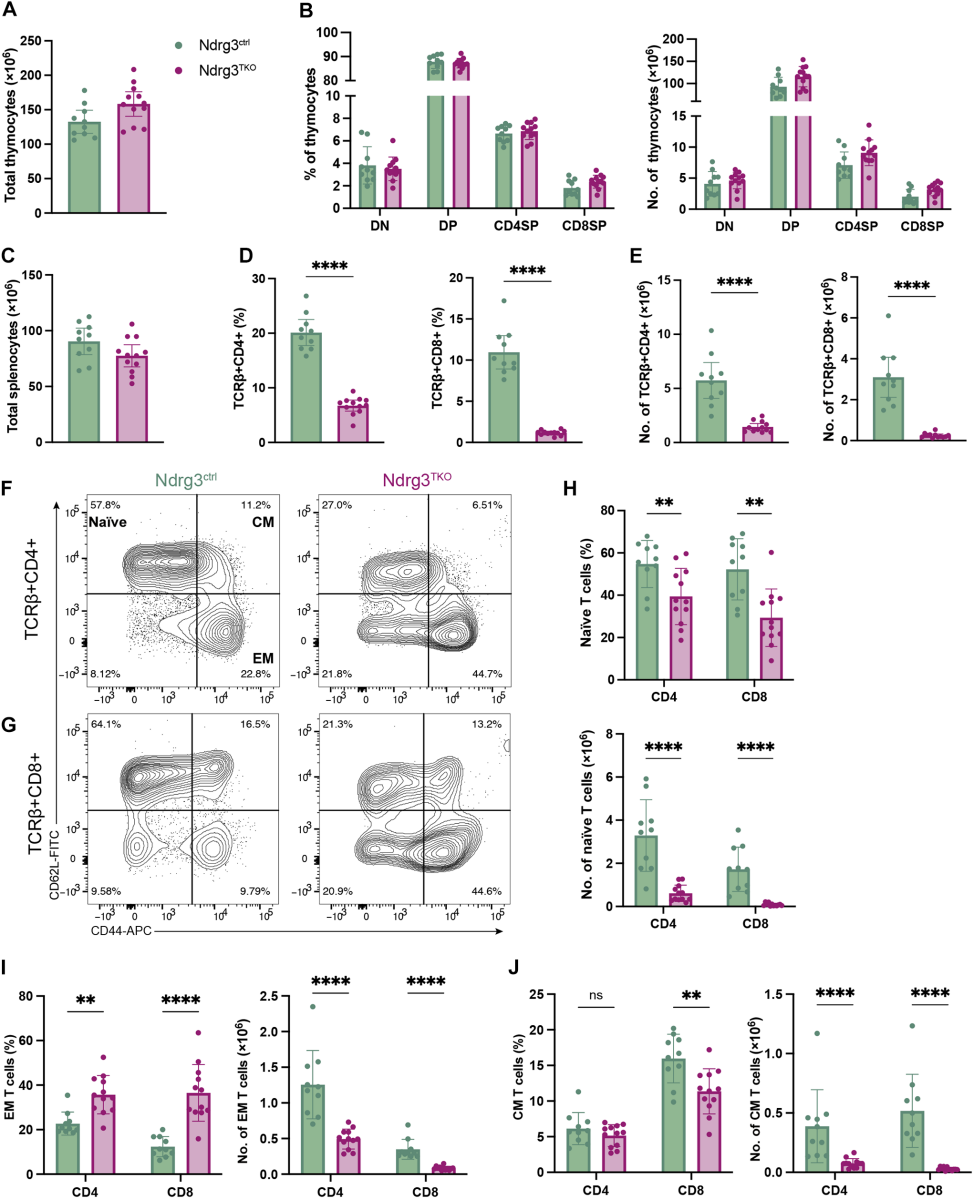
Ndrg3 is indispensable for peripheral T cells. Total thymocyte numbers from Ndrg3^ctrl^ (*Ndrg3^+/fl^;Lck-*cre+) and Ndrg3^TKO^ (*Ndrg3^fl/fl^;Lck*-cre+) mice are shown in (**A**). Proportions and cell numbers of DN, DP, CD4SP, and CD8SP thymocytes are depicted in (**B**). Total splenocyte numbers from Ndrg3^ctrl^ and Ndrg3^TKO^ are shown in (**C**). Proportions (**D**) and cell numbers (**E**) of TCRβ+CD4+ and TCRβ+CD8+ cells in the spleen. Representative gating of naïve (CD62L+CD44^low^), CM (CD62L+CD44^high^), and EM (CD62L−CD44^high^) subsets in TCRβ+CD4+ (**F**) and TCRβ+CD8+ (**G**) splenocytes. Proportions and cell numbers of naïve (**H**), EM (**I**), and CM (**J**) TCRβ+CD4+ and TCRβ+CD8+ splenocytes are quantified. Scatter dot plots are presented as the means ± SD with each symbol representing an individual mouse. Data collected from ≥2 experiments. Comparison between groups was calculated with unpaired *t* tests. **P < 0.01, ****P < 0.0001, and nonsignificant (ns) data indicate *P* > 0.05.

To investigate the role of Ndrg3 in T cell biology, we depleted Ndrg3 at an early stage of T cell development using a proximal Lck-Cre driver [pLck-Cre; (*39*)]. Western blot analysis confirmed that Ndrg3 was expressed in the thymus (fig. S1A) and that Ndrg3 was efficiently depleted in thymocytes from Ndrg3fl/fl; pLck-Cre+ mice (fig. S1B). Despite efficient deletion of Ndrg3, thymocyte development was largely normal in Ndrg3fl/fl; pLck-Cre+ (hereafter Ndrg3^TKO^) when compared to control mice (Ndrg3+/fl; pLck-Cre+, Ndrg3^ctrl^). Total thymocyte counts (Fig. 1A) and the proportions and total numbers of double-negative (DN), double-positive (DP), CD4 single-positive (CD4SP), and CD8 single-positive (CD8SP) subsets were unchanged in Ndrg3^TKO^ thymi relative to controls (Fig. 1B). Moreover, detailed examination of DP cells revealed that Ndrg3^TKO^ cells progressed normally through the DP1 to DP3 maturation stages (fig. S1, C to E) (*40*) and both the levels of CD69 expression (fig. S1F) and proportions of DP cells undergoing positive selection were unchanged (fig. S1G). Ndrg3^ctrl^ and Ndrg3^TKO^ mice also exhibited equivalent proportions of active caspase-3–positive cells (fig. S1H), indicating no major changes in cells undergoing negative selection (*41*). Likewise, examination of CD4SP and CD8SP thymocytes failed to reveal any changes in the expression of late maturation markers (fig. S1, I and J) (*42*). Therefore, on the basis of surface marker expression, the development of DN, DP, and SP subsets appeared to be largely normal in Ndrg3-deficient mice.

### Ndrg3 is necessary for the establishment of peripheral T cell populations

Analysis of peripheral T cell populations, however, revealed a different picture. Although total splenocyte numbers were similar (Fig. 1C), the proportions and absolute numbers of CD4+ and CD8+ T cells were markedly reduced in Ndrg3^TKO^ mice compared to controls (Fig. 1, D and E; gated as depicted in fig. S2, A and B). Staining for the maturation markers CD44 and CD62L revealed significant changes among naïve (CD44^low^CD62L+), effector memory (EM; CD44^high^CD62L−), and central memory (CM; CD44^high^CD62L+) T cell subsets in Ndrg3^TKO^ spleens (Fig. 1, F to J), and both CD4+ (Fig. 1F) and CD8+ (Fig. 1G) subsets were affected. In terms of absolute cell counts, all subsets (naïve, EM, and CM) were decreased in Ndrg3^TKO^ mice relative to controls (Fig. 1, H to J). The distribution of the remaining T cells present in Ndrg3^TKO^ mice was significantly skewed; naïve cell proportions were strongly reduced (Fig. 1H), while EM T cell proportions increased (Fig. 1I). These changes in naïve- and EM-type cells were observed in both the CD4+ and CD8+ subsets but were especially prominent among CD8+ cells. Proportions of CM cells were decreased among CD8+ T cells from Ndrg3^TKO^ mice; however, no change was observed among the corresponding CD4+ population (Fig. 1J). Notably, although regulatory T cells (T_regs_) were found in normal numbers and proportions in Ndrg3^TKO^ thymi (fig. S2, C and D), the proportion of Foxp3+CD25+ T_regs_ in the spleen was slightly increased within the total CD4+ T cell population (fig. S2E). Nonetheless, the total numbers of peripheral T_regs_ were reduced (fig. S2E). Collectively, these data indicated that Ndrg3 has an important role in regulating peripheral CD4+ and CD8+ T cell homeostasis and that CD8+ T cells are more sensitive to Ndrg3 loss than CD4+ cells (fig. S2G).

### Loss of naïve T cell quiescence in Ndrg3^TKO^ mice

**Fig. 2.**
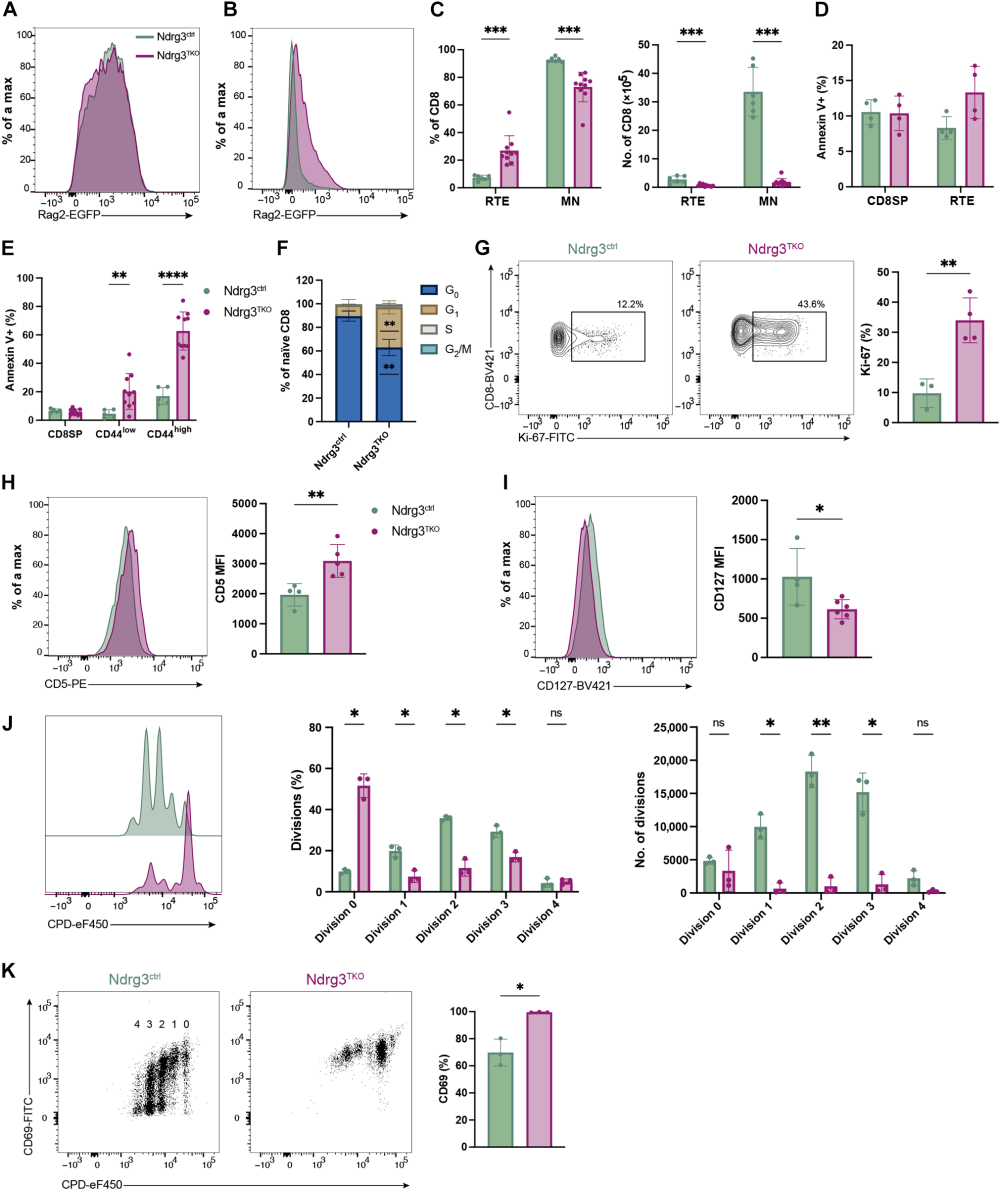
Ndrg3 deficiency impairs T cell homeostasis and results in increased apoptosis. Representative histograms of EGFP expression from Rag2-EGFP+Ndrg3^ctrl^ (*Ndrg3^+/fl^;Lck*-cre+;Rag2-EGFP) and Ndrg3^TKO^ (*Ndrg3^fl/fl^;Lck*-cre+;Rag2-EGFP) mice in CD8SP thymocytes and TCRβ+CD8+ splenocytes are shown in (**A**) and (**B**), respectively. Proportions and cell numbers of RTE and mature naïve (MN) T cells are quantified in (**C**). Proportions of annexin V+ cells among CD8SP and RTE are presented in (**D**). Annexin V+ proportions in CD8SP thymocytes and CD44^low^ and CD44^high^ TCRβ+CD8+ splenocytes from Ndrg3^ctrl^ and Ndrg3^TKO^ mice are depicted in (**E**). Cell cycle analysis of TCRβ+CD8+CD62L+CD44^low^ cells is shown in (**F**). G_0_ (Ki-67^neg^DAPI^low^), G_1_ (Ki-67^pos^DAPI^low^), S (Ki-67^pos^DAPI^intermidiate^), and G_2_/M (Ki-67^pos^DAPI^high^). Representative gating of Ki-67+ cells in TCRβ+CD8+CD62L+CD44^low^ population is shown and quantified in (**G**). Flow cytometry histograms and quantifications of CD5 and CD127 expression in TCRβ+CD8+CD62L+CD44^low^ cells are shown in (**H**) and (**I**), respectively. Naïve CD8 (CD8+CD62L+CD44^low^) T cells (8 × 10^4^) from Ndrg3^ctrl^ and Ndrg3^TKO^ mice were labeled with cell proliferation dye (CPD) and stimulated with anti-CD3– and anti-CD28–coated beads for 48 hours at 37°C. Depicted in (**J**) are flow cytometry histograms illustrating division peaks (left panel) and quantifications of dividing cell proportions (middle panel) and numbers (right panel) after 48 hours of culture. Representative gating and proportions of CD69+ cells in proliferating naïve T cells are presented in (**K**). Scatter dot plots are presented as the means ± SD with each symbol representing an individual mouse. Data were collected from ≥2 experiments. Comparison between groups was calculated using unpaired *t* tests. *P < 0.05, **P < 0.01, ***P < 0.001, ****P < 0.0001, and ns data indicate *P* > 0.05.

As the effects of Ndrg3 deficiency were more prominent in CD8+ T cells than in CD4+ T cells, we focused on the CD8+ subset for the remainder of this study. Staining with a panel of antibodies against diverse TCR Vα and Vβ elements demonstrated that while TCR repertoires were similar among CD8SP cells from Ndrg3^TKO^ and Ndrg3^ctrl^ mice, Vα and Vβ usage was skewed in the spleen of Ndrg3^TKO^ mice (fig. S2, H to O). These results once again indicated that the phenotypic consequences of Ndrg3 loss manifest during the transition from thymic to peripheral T cell development. Therefore, to determine the cause of peripheral CD8 T cell reduction upon Ndrg3 loss, we next analyzed RTE populations using a Rag2-EGFP reporter (*43*). We detected no difference in enhanced green fluorescent protein (EGFP) expression levels between CD8SP cells isolated from the thymi of Rag2-EGFP–transgenic Ndrg3^ctrl^ and Ndrg3^TKO^ mice (Fig. 2A), indicating the normal export of RTEs, as EGFP^lo/−^ cells did not accumulate in Ndrg3-deficient mice. Conversely, examination of splenocytes showed that EGFP+ RTEs accounted for a much greater proportion of the CD8+ T cells present in Ndrg3^TKO^ mice than in Ndrg3-sufficient controls (Fig. 2, B and C). In control mice, RTEs accounted for ~7% of the total CD8+ T cell pool; however, among the few CD8+ T cells present in Ndrg3^TKO^, more than a quarter of the cells were EGFP+ RTEs (Fig. 2C). Staining with annexin V failed to reveal any significant changes in rates of apoptosis among CD8SP thymocytes or RTEs in the spleen (Fig. 2D), suggesting that the diminished number of T cells in Ndrg3^TKO^ mice is not due to the immediate loss of RTEs themselves but rather due to defective peripheral T cell maturation. In support of this, we observed an increase of annexin V+ T cells from Ndrg3^TKO^ mice as they matured: Unlike CD8SP thymocytes and RTEs, CD8+CD44^low^ cells exhibited enhanced rates of apoptosis compared to controls, and this effect was even more prominent in the CD8+CD44^high^ compartment (Fig. 2E). Given that T cell homeostasis requires balanced levels of cell replacement and loss, we next examined rates of homeostatic proliferation among the peripheral naïve CD8+ T cells in Ndrg3^TKO^ mice. Cell cycle analysis revealed that the loss of Ndrg3 in CD8+ T cells stimulated the progression of Ndrg3^TKO^ cells into the cell cycle, as we observed that the proportion of naïve CD8+ T cells in the G_1_ phase was significantly increased relative to controls (Fig. 2F). Intracellular staining for the proliferation marker Ki-67 supported this conclusion and revealed that approximately one-third of the CD62L+CD44^low^CD8+ T cells in Ndrg3^TKO^ were Ki-67+ (Fig. 2G). The naïve CD8+ T cell population in Ndrg3-deficient mice therefore appears to have lost its quiescent state and is characterized by increased rates of both apoptosis and proliferation. Consequently, we aimed to find the cause of enhanced rates of proliferation in naïve Ndrg3-deficient T cells, which occurred even in the absence of intentional antigen stimulation.

### Ndrg3 maintains the balance of homeostatic signals required for the survival of peripheral T cells

Homeostatic proliferation is largely dependent on tonic TCR signals and IL-7 signaling (*4*). We first questioned whether tonic signaling is maintained in Ndrg3-deficient mice. As CD5 expression has been previously described to reflect the intensity of tonic signaling (*44*), and high-avidity TCRs have a survival advantage under homeostatic conditions (*45*), we used CD5 expression to assess the tonic signaling strength and TCR avidity of CD8+ T cells in Ndrg3-deficient mice (*46*, *47*). No difference in CD5 levels was detected between Ndrg3^ctrl^ and Ndrg3^TKO^ CD8SP thymocytes (fig. S3A). In contrast, naïve CD8+ T cells in the spleen from Ndrg3^TKO^ displayed increased CD5 expression compared to controls (Fig. 2H), indicating that the surviving CD8+ cells in Ndrg3-deficient mice may be expressing high-avidity TCRs capable of enhanced tonic signaling. Furthermore, as IL-7 is a central regulator of T cell survival and homeostasis (*13*), we examined the expression of CD127 (IL-7Rα) by Ndrg3-deficient T cells. In the thymus, Ndrg3^ctrl^ and Ndrg3^TKO^ CD8SP thymocytes expressed equivalent levels of CD127 (fig. S3B); however, CD127 was significantly reduced in naïve Ndrg3-deficient CD8+ T cells isolated from the spleen (Fig. 2I). These differences were the most pronounced among naïve Ndrg3-deficient CD8+ T cells; Ndrg3^TKO^ CM cells exhibited increased Ki-67 and CD5 expression but normal levels of CD127, and Ndrg3^TKO^ EM cells displayed levels of Ki-67, CD5, and CD127 similar to controls (fig. S3, C to H). It has been previously described that cell surface IL-7 receptor levels can be regulated by transcriptional mechanisms (*48*) or by ligand binding and receptor internalization (*49*). To test whether Ndrg3-deficient T cells express diminished surface CD127 because of an intrinsic defect or because of increased ligand-induced internalization, we sorted naïve (CD8+CD44^low^CD62L+) splenocytes from Ndrg3^ctrl^ and Ndrg3^TKO^ mice (fig. S4A) and cultured them overnight in media alone in the absence of IL-7. Under these conditions, surface expression of CD127 normalized (fig. S4B), demonstrating that there is no intrinsic defect in the ability of Ndrg3-deficient cells to express the IL-7 receptor. This finding indicates that low surface CD127 levels among CD8+ T cells from Ndrg3-deficient mice are due to continuous IL-7 stimulation and receptor internalization. Furthermore, when stimulated with IL-7 in culture overnight, Ndrg3^ctrl^ and Ndrg3^TKO^ CD8+ T cells exhibited equivalent levels of signal transducers and activators of transcription 5 phosphorylation (fig. S4C), indicating that Ndrg3-deficient cells are capable of responding to IL-7 despite the low expression of its receptor in vivo. Collectively, these results indicate that the surviving naïve CD8+ T cells in Ndrg3^TKO^ are skewed toward high CD5 expression and are exposed to chronic IL-7 signaling because of reduced consumption of this cytokine under lymphopenic conditions (*50*).

### Diminished proliferation and augmented cytokine secretion upon stimulation of Ndrg3-deficient T cells

Having demonstrated that Ndrg3-deficient cells lose quiescence and exhibit increased susceptibility to apoptosis, we next examined whether Ndrg3-deficient cells exhibited similar defects when activated. Preliminary experiments revealed that Ndrg3 protein levels increased in wild-type T cells 48 hours after activation with anti-CD3/CD28 (fig. S4D), and we therefore asked whether loss of Ndrg3 affects the response of naïve CD8+ T cells to antigen activation. To investigate this question, we isolated Ndrg3^TKO^ naïve CD8+ cells and performed in vitro proliferation assays. We found that Ndrg3 deficiency impairs the T cell response to activation as most Ndrg3^TKO^ cells failed to divide when stimulated with αCD3/CD28–coated beads (Fig. 2J). In contrast, Ndrg3^ctrl^ cells underwent one to four divisions after 48-hour stimulation. Curiously, although naïve T cells lacking Ndrg3 did not proliferate efficiently, they were nevertheless activated, as revealed by the high expression of the early activation marker CD69 at all division stages (Fig. 2K). CD8+ T cells from Ndrg3^ctrl^ mice also expressed high levels of CD69; however, this activation marker was gradually down-regulated as cells proliferated. Conversely, Ndrg3^TKO^ exhibited little down-regulation of CD69, even among the few cells that managed to divide. Notably, although Ndrg3^TKO^ cells proliferated less than controls in response to anti-CD3/CD28 stimulation, they secreted much higher levels of the cytokines interferon-γ (IFN-γ) and tumor necrosis factor–α (TNF-α) (fig. S4E). Naïve, Ndrg3-deficient T cells also exhibited defective proliferative responses when pharmacologically activated with phorbol 12-myristate 13-acetate (PMA)/ionomycin (fig. S4F), demonstrating that this defect is not unique to direct TCR activation elicited by αCD3/CD28. In conclusion, although Ndrg3-deficient naïve cells could be readily activated, they responded abnormally to antigenic stimulation, in that they failed to expand in number, and secreted exaggerated levels of pro-inflammatory cytokines compared to the control cells.

### High-affinity TCR promotes the survival of naïve T cells in Ndrg3-deficient mice

**Fig. 3.**
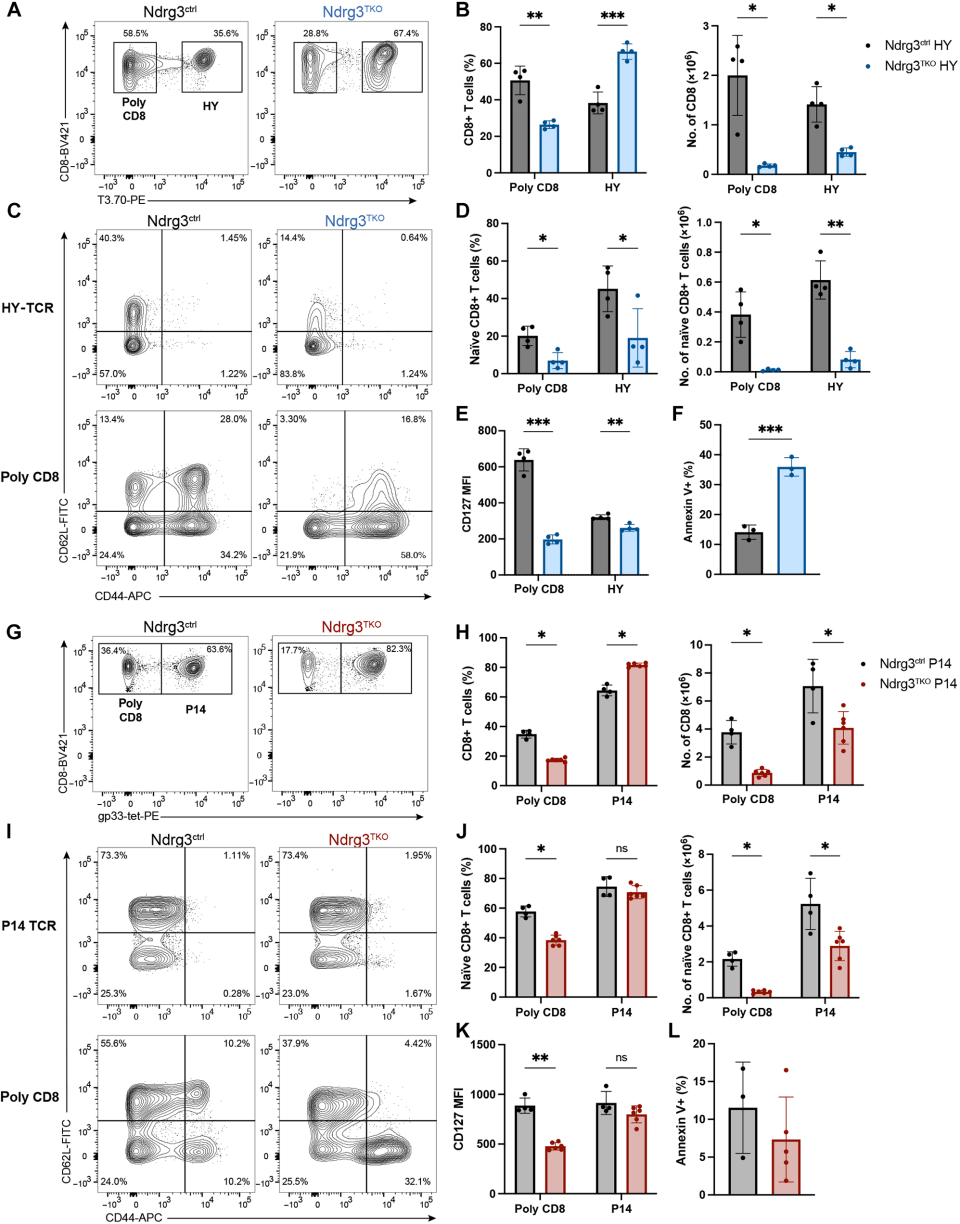
High-affinity P14 TCR partially rescues naïve T cell populations in Ndrg3^TKO^ mice. Representative gating of polyclonal CD8 (CD8+T3.70−) and HY CD8 (CD8+T3.70+) cells among splenocytes harvested from 6-week-old female Ndrg3^ctrl^ HY (*Ndrg3^+/fl^;Lck*-cre+;HY) and Ndrg3^TKO^ HY (*Ndrg3^fl/fl^;Lck*-cre+;HY) mice is presented in (**A**). Proportions and numbers of CD8 T cells from the spleen are depicted in (**B**). Representative gating of naïve CD8 (CD8+CD62L+CD44^low^) splenocytes from Ndrg3^ctrl^ HY and Ndrg3^TKO^ HY mice is shown in (**C**), and proportions and numbers are quantified in (**D**). Median fluorescence intensity (MFI) of CD127 in CD8+ splenocytes is presented in (**E**). Proportions of annexin V+ cells in HY CD8 splenocytes are shown in (**F**). Representative gating of polyclonal CD8 (CD8+gp33-tetramer−) and P14 CD8 (CD8+gp33-tetramer+) splenocytes from Ndrg3^ctrl^ P14 (*Ndrg3^+/fl^;Lck*-cre+;P14) and Ndrg3^TKO^ P14 (*Ndrg3^fl/f^;Lck*-cre+;P14) mice is illustrated in (**G**). Proportions and cell numbers of polyclonal CD8 and P14-expressing CD8 splenocytes are quantified in (**H**). Representative flow cytometry gating of naïve CD8 (CD8+CD62L+CD44^low^) polyclonal and P14 CD8 cells is shown in (**I**) and quantified in (**J**). MFI of CD127 is shown in (**K**). The percentage of annexin V+ cells in P14 CD8 splenocytes is presented in (**L**). HY-TCR data were collected from female mice only. All data originate from ≥2 experiments. Scatter dot plots are presented as the means ± SD with each symbol representing an individual mouse. Comparison between groups was calculated with unpaired *t* tests. *P < 0.05, **P < 0.01, ***P < 0.001, and ns data indicate *P* > 0.05.

Our results so far indicated that Ndrg3-deficient CD8+ T cells were able to egress the thymus but failed to efficiently establish themselves in the immune periphery. Intriguingly, increased CD5 expression in Ndrg3^TKO^ (Fig. 2H) suggested that surviving naïve cells in the periphery were enriched for the expression of high-avidity TCRs; however, it remained possible that the increased CD5 levels were instead the result of their loss of quiescence among Ndrg3-deficient cells. Therefore, to investigate whether the change in the avidity of TCR could be the cause of naïve cell loss in Ndrg3^TKO^ naïve cells, we used two TCR-transgenic models on the basis of the expression of either a low-avidity HY-TCR transgene (*51*) or high-avidity P14 transgene (*52*). We first tested how the low-avidity HY-TCR influenced the phenotype of Ndrg3-deficient peripheral T cells. In this model, we used a clonotypic antibody to divide peripheral T cells from female, HY-TCR–transgenic, Ndrg3^ctrl^ or Ndrg3^TKO^ mice into populations that expressed the transgenic TCR and cells that expressed polyclonal, endogenously rearranged TCRs (Fig. 3A). HY-TCR–expressing cells were proportionally enriched in Ndrg3^TKO^;HY-TCRTg mice relative to Ndrg3^ctrl^;HY-TCRTg controls; nonetheless, the expression of the TCR transgene could not restore the CD8+ T cell population in Ndrg3-deficient mice (Fig. 3, A and B). Moreover, examination of CD44 and CD62L expression revealed that the HY-TCR did not rescue the naïve T cell population in Ndrg3-deficient animals (Fig. 3, C and D), and as previously reported (*15*), expression of this low-avidity TCR failed to support differentiation of EM cells under homeostatic conditions (Fig. 3C). Polyclonal CD8+ T cells from the same mice, however, revealed a strong skewing toward an EM phenotype in the absence of Ndrg3 (Fig. 3C), as was seen previously in non–TCR-transgenic, Ndrg3-deficient mice (Fig. 1I). Furthermore, expression of the HY-TCR failed to restore normal levels of the IL-7 receptor expression (Fig. 3E), and Ndrg3-deficient, HY-TCR–expressing cells still exhibited increased rates of apoptosis relative to controls (Fig. 3F). Contrastingly, in male mice, which express the cognate antigen recognized by the HY-TCR, Ndrg3 deficiency had little effect on HY-TCR–expressing CD8+ T cell development and maturation. The thymi of both Ndrg3TKO;HY-TCRTg and Ndrg3ctrl;HY-TCRTg male mice exhibited the low overall cellularity (fig. S5A) and proportions of DP thymocytes (fig. S5B), as previously reported (*51*). The total numbers of DN, DP, CD4SP, and CD8SP thymocytes were similar in both Ndrg3ctrl and Ndrg3TKO mice expressing the HY-TCR (fig. S5B), indicating that Ndrg3 deficiency did not impair negative selection processes in the thymus of male mice. As clonal deletion is not complete in male HY-TCR–transgenic mice, we further investigated peripheral CD8+ T cell populations in the spleens of Ndrg3TKO;HY-TCRTg and Ndrg3ctrl;HY-TCRTg mice to see whether Ndrg3 influenced peripheral maturation in a setting of chronic self-stimulation. In male TCR-transgenic mice, >90% of splenic CD8+ T cells expressed the HY-TCR, and few polyclonal T cells were present (fig. S5, C and D), regardless of Ndrg3 status. Intriguingly, we observed no differences in the proportions or numbers of naïve, CM, or EM subsets (fig. S5, E to C) between Ndrg3-sufficient and Ndrg3-deficient male, HY-TCR–transgenic mice. These findings demonstrated that while Ndrg3 was important for the survival of low-avidity HY-TCR–expressing cells in female mice, it was dispensable in male mice where cells bearing the same TCR receive chronic, high-avidity stimulation.

**Fig. 4.**
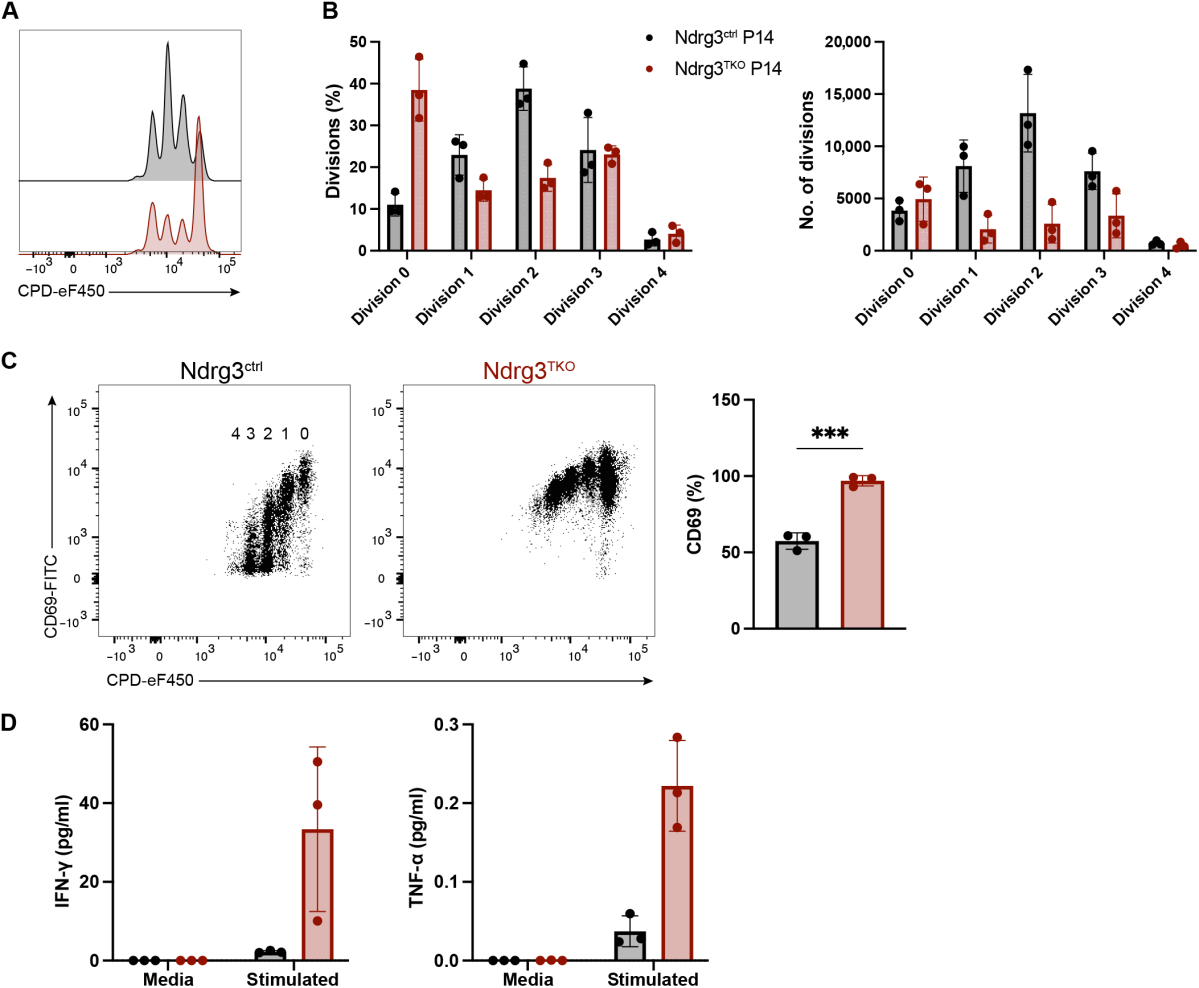
Ndrg3-deficient naïve CD8+ T cells expressing a high-avidity TCR fail to respond normally to activation. Purified 8 × 10^4^ naïve CD8 splenocytes (CD8+CD62L+) from Ndrg3^ctrl^ P14 and Ndrg3^TKO^ P14 mice were labeled with CPD and incubated with anti-CD3– and anti-CD28–coated beads at 37°C for 48 hours. Representative flow cytometry histograms illustrating division peaks are shown in (**A**). Dividing cell proportions (left panel) and numbers (right panel) are quantified in (**B**). Expression of CD69 in proliferating cells is presented and quantified in (**C**). Concentrations (pg/ml) of IFN-γ and TNF-α released to cell culture medium by proliferating (stimulated) or unstimulated (media) P14 naïve CD8 T cells are shown in (**D**). Scatter dot plots are presented as the means ± SD with each symbol representing an individual mouse. Data were collected from three experiments. Comparison between groups was calculated with unpaired *t* tests. ***P < 0.001.

To test whether this was also true in the case of a high-avidity TCR, which, unlike the HY-TCR in male mice, is not subject to partial clonal deletion in the thymus, we next expressed the high-avidity P14 TCR (*52*) in an Ndrg3-deficient background. In this instance, we used a gp33 tetramer to distinguish cells expressing the transgenic TCR and those expressing an endogenous TCR (Fig. 3G). Comparisons of these populations revealed that the P14 TCR conferred a survival advantage to Ndrg3-deficient T cells. We observed a partial recovery of total CD8+ T cell numbers (Fig. 3H) in these mice, and expression of the P14 TCR allowed normal proportions of naïve T cells to develop in Ndrg3-deficient mice, although absolute numbers of naïve, P14-expressing cells were still reduced relative to Ndrg3-sufficient controls (Fig. 3, I and J). This rescue effect was cell intrinsic, as polyclonal CD8+ T cells isolated from Ndrg3-deficient, TCR-transgenic mice exhibited reduced proportions of naïve cells and increased proportions of EM-type cells, as we previously observed (Fig. 3, I and J). In addition, expression of the P14 TCR was also sufficient to restore CD127 levels in Ndrg3^TKO^ cells (Fig. 3K) and suppress apoptosis, as measured by annexin V staining (Fig. 3L). Collectively, these data demonstrate that high-avidity TCRs can partially compensate for the loss of Ndrg3 and suggest that Ndrg3 acts to fine tune TCR tonic signaling under homeostatic conditions. Given that expression of the P14 TCR was able to substantially rescue the naïve T cell population in Ndrg3-deficient mice, we further tested whether these cells could respond to antigen activation. When stimulated in vitro using αCD3/CD28–coated beads, naïve P14-expressing, Ndrg3-deficient CD8+ T cells proliferated less than Ndrg3-sufficient controls (Fig. 4, A and B). As was the case for nontransgenic cells, many P14+;Ndrg3^TKO^ cells failed to expand; however, these cells were nevertheless activated (Fig. 4C) and secreted much higher levels of IFN-γ and TNF-α than Ndrg3-sufficient controls (Fig. 4D). In contrast, however, we found that when naïve P14-expressing, Ndrg3-deficient CD8+ T cells were strongly stimulated with gp33 peptide–loaded splenocytes, they proliferated similarly to control cells (fig. S6, A and B), although they still secreted abnormal amounts of IFN-γ and TNF-α (fig. S6C). These results further indicate that high TCR avidity and signal strength can at least partially compensate for the absence of Ndrg3 in naïve CD8+ T cells.

### Ndrg3 is required for the normal expression of Eomes and Tbet

**Fig. 5.**
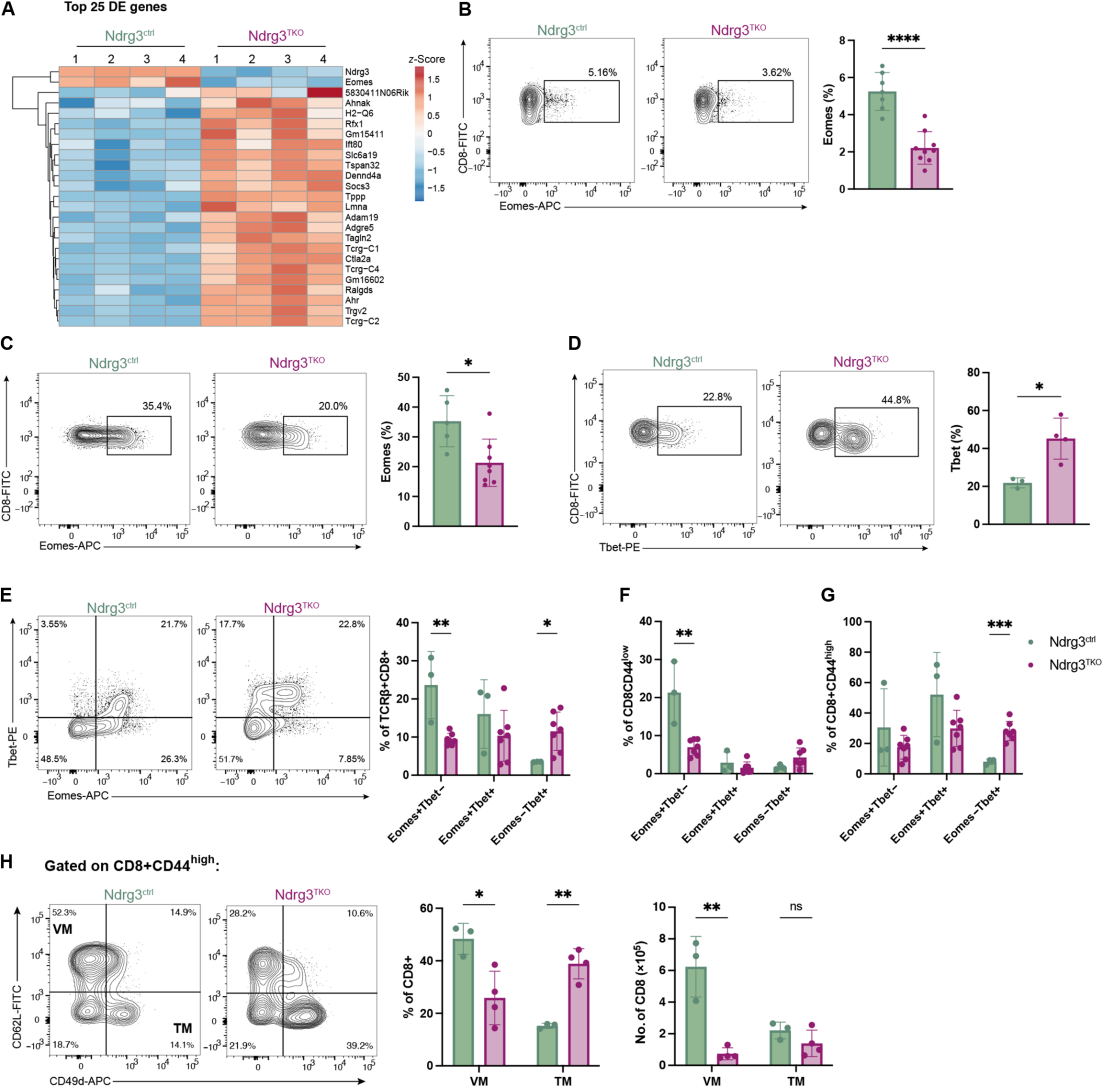
Ndrg3-deficient CD8+ T cells exhibit changes in the expression of the T-box transcription factors, Eomes and Tbet. CD8SP TCRβ+MHC1^high^ thymocytes were sorted and submitted for bulk RNA-seq analysis. The top 25 differentially expressed (DE) genes between Ndrg3^TKO^ and Ndrg3^ctrl^ are shown in (**A**). The heatmap shows the row *z*-score of counts. Four independent biological replicates were analyzed. Flow cytometry analysis of Eomes expression in CD8SP thymocytes from Ndrg3^ctrl^ and Ndrg3^TKO^ mice is illustrated in (**B**). Representative flow cytometry gating and quantification of Eomes (**C**) and Tbet (**D**) expression in TCRβ+CD8+ splenocytes. Flow cytometry plots representing the gating strategy for the coexpression of Eomes and Tbet in TCRβ+CD8+ splenocytes (**E**) and quantification of proportions in total TCRβ+CD8+ (E), TCRβ+CD8+CD44^low^ (**F**), and TCRβ+CD8+CD44^high^ (**G**). Representative gating strategy and proportions of CD8+ VM (CD8+CD62L+CD44^high^CD49d^low^) and TM (CD8+CD62L+CD44^high^CD49d^high^) splenocytes are presented in (**H**). Scatter dot plots originate from ≥2 experiments and are presented as the means ± SD with each symbol representing an individual mouse. Comparison between groups was calculated with unpaired *t* tests. *P < 0.05, **P < 0.01, ***P < 0.001, ****P < 0.0001, and ns data indicate *P* > 0.05.

Our studies indicated that Ndrg3-deficient CD8+ T cells were defective in transitioning from CD8SP thymocytes into mature naïve peripheral T cells. To identify the early event(s) behind this defect, we performed RNA sequencing (RNA-seq) on MHC1+ mature CD8SP thymocytes sorted from Ndrg3^ctrl^ and Ndrg3^TKO^ mice (fig. S7A). Differential gene expression analysis revealed 205 down-regulated genes and 298 up-regulated genes in Ndrg3^TKO^ cells relative to controls (fig. S7B and data S1), with a number of pathways dysregulated (fig. S7, C and D). Analysis of the top 25 differentially expressed genes revealed that the two most down-regulated genes in CD8SP thymocytes from Ndrg3^TKO^ mice were *Ndrg3* and the T-box transcription factor *Eomes* (Fig. 5A). Intracellular staining of CD8SP thymocytes confirmed the down-regulation of Eomes in Ndrg3-deficient cells (Fig. 5B). Given that Eomes has multiple roles in modulating T cell differentiation and function (*53*–*56*), we further investigated Eomes expression in peripheral CD8+ T cells and observed that the frequency of Eomes-expressing cells was also reduced among CD8+ T cells from the spleen of Ndrg3^TKO^ mice (Fig. 5C). As Eomes and Tbet are often expressed in reciprocal patterns (*57*), we investigated whether Tbet expression was also altered in Ndrg3^TKO^ mice. In contrast to Eomes, we found that proportions of Tbet+ cells were significantly increased in Ndrg3^TKO^ mice compared to controls (Fig. 5D). To better characterize the interplay of Eomes and Tbet expression, we performed additional costaining and found that Ndrg3-deficient CD8+ T cells exhibited an overall decrease in the proportion of Eomes–single-positive cells and an increase in the proportion of Tbet–single-positive cells, while proportions of Eomes+Tbet+–double-positive cells were unchanged (Fig. 5E). These changes were primarily due to a reduction in Eomes–single-positive cells in the CD8+CD44^low^ fraction (Fig. 5F) and an increase of Tbet–single-positive cells within the CD8+CD44^high^ fraction (Fig. 5G) of Ndrg3^TKO^ mice. Given that T cell factor 1 (Tcf1) is an important regulator of Eomes expression (*58*), we analyzed Tcf1 expression in DP and CD8SP thymocytes, as well as in splenic CD8+ T cells, but found no differences between Ndrg3-deficient and control mice (fig. S7, E to G), demonstrating that the reduction of Eomes+ cells in Ndrg3^TKO^ mice is not due to diminished expression of Tcf1. Eomes is expressed in several CD8+ T cell subsets and is strongly associated with memory-like cells, including virtual memory (VM) cells (*59*), which can make up a substantial fraction of the peripheral T cell pool. We therefore asked whether VM populations were altered in Ndrg3-deficient mice and used flow cytometry to identify VM (CD44^high^CD49d^low^CD62L+) and true memory (TM; CD44^high^CD49d^high^CD62L+) cells (*60*). Compared to Ndrg3^ctrl^ mice, VM proportions were reduced among CD8+ T cells from Ndrg3^TKO^ (Fig. 5H), consistent with the decreased frequency of Eomes expression in the absence of Ndrg3.

### Ndrg3-deficient cells respond poorly to IL-4 stimulation

**Fig. 6.**
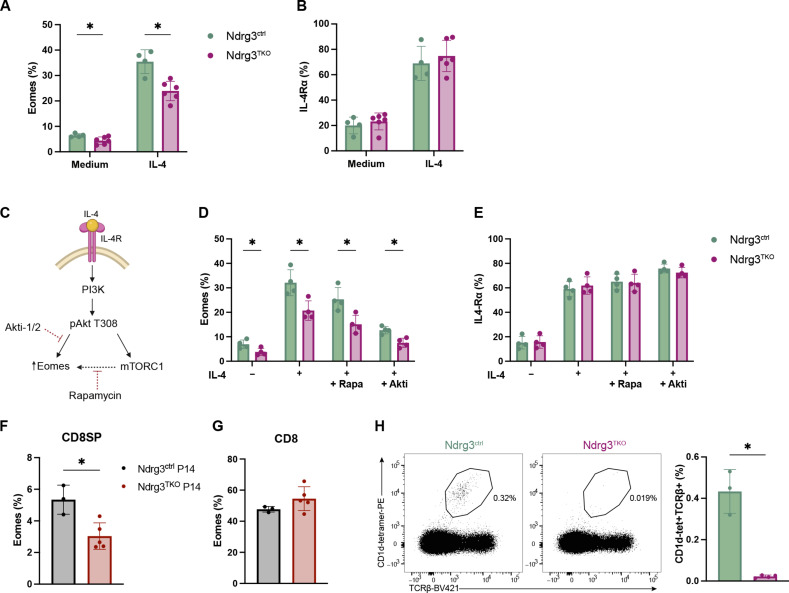
Ndrg3 is required for efficient responses to IL-4. Sort-purified CD8SP thymocytes (2 × 10^5^) from Ndrg3^ctrl^ and Ndrg3^TKO^ mice were plated in 200 μl of IMDM and 10% FCS supplemented with IL-4 (20 ng/ml), cultured for 20 hours at 37°C, and subsequently analyzed by flow cytometry for the induction of Eomes and IL-4Rα. Quantification of Eomes and IL-4Rα expression is shown in (**A**) and (**B**), respectively. A drawing of IL-4-Akt–dependent Eomes induction in CD8SP thymocytes is shown in (**C**). The illustration was generated on the basis of previously published experimental evidence (*61*) and created with BioRender.com. CD8SP thymocytes were sorted and stimulated with IL-4 the same way as in (A) with addition of 25 nM rapamycin or 5 μM Akti. After 20 hours of incubation, proportions of Eomes and IL-4Rα were measured and are shown in (**D**) and (**E**), respectively. Proportions of Eomes expression in total CD8SP thymocytes and CD8+ splenocytes from Ndrg3^ctrl^ P14 and Ndrg3^TKO^ P14 mice are quantified in (**F**) and (**G**), respectively. Representative gating and quantification of thymic NKT cells (CD1d-tetramer+TCRβ+) from Ndrg3^ctrl^ Ndrg3^TKO^ mice are depicted in (**H**). Scatter dot plots originate from ≥2 experiments and are presented as the means ± SD with each symbol representing an individual mouse. Comparison between groups was calculated with unpaired *t* tests. *P < 0.05.

IL-4 plays a central role in inducing Eomes expression (*61*–*63*). Therefore, we next examined whether IL-4 responses were compromised in Ndrg3-deficient CD8+ T cells. To this end, we sorted CD8SP MHC1+ thymocytes from Ndrg3^ctrl^ and Ndrg3^TKO^ mice and cultured them overnight in media with or without IL-4 (fig. S8A). Incubation with IL-4 was able to increase the frequency of Eomes expression in both Ndrg3^ctrl^ and Ndrg3^TKO^ cells; however, the induction was lower in Ndrg3-deficient cells (Fig. 6A). This diminished response to IL-4 could not be attributed to reduced receptor expression by Ndrg3^TKO^ cells, as no differences in surface levels of IL-4Rα and the common γ chain (CD132) were detected between Ndrg3^TKO^ and Ndrg3^ctrl^ cells (fig. S8, B to G). IL-4 is known to induce the expression of its own receptor (*61*, *64*), and our experiments revealed that both Ndrg3^ctrl^ and Ndrg3^TKO^ cells up-regulated IL-4Rα to the same level after overnight IL-4 treatment (Fig. 6B). Therefore, in Ndrg3-deficient cells, the ability of IL-4 to induce receptor expression was intact, but the ability to induce Eomes expression was compromised. IL-4–mediated induction of Eomes has been demonstrated to rely on both Akt and mammalian target of rapamycin complex 1 signaling, with phosphorylation of Akt reported to play the dominant role (Fig. 6C) (*61*). We therefore used inhibitors to determine whether the defective IL-4–induced up-regulation of Eomes observed in Ndrg3^TKO^ could be attributed to defects in either the Akt or mammalian target of rapamycin complex 1 pathways. In line with a previous report (*61*), we found that in control cells, rapamycin had only a minor effect on the induction of Eomes in response to IL-4 treatment; however, inhibition of Akt with its selective inhibitor (Akti) almost completely blocked Eomes induction (Fig. 6D). Inhibitor treatment of Ndrg3^TKO^ cells revealed similar trends. As observed previously, overall induction of Eomes was low in Ndrg3-deficient cells, and this was further impaired by treatment with rapamycin or Akti. These results indicate that Ndrg3 influences IL-4 signaling upstream of pAkt. Notably, although rapamycin and Akti treatments inhibited Eomes induction by IL-4, they did not impair the ability of either control or conditional knockout cells to up-regulate IL-4Rα (Fig. 6E). As we had previously shown that expression of the P14 TCR was able to partially rescue T cell development and naïve T cell populations in Ndrg3^TKO^ mice, we next tested whether Eomes levels were restored under these conditions. We found that although the frequency of Eomes+ cells was still reduced among CD8SP thymocytes in Ndrg3^TKO^;P14-transgenic mice relative to controls (Fig. 6F), the proportion of Eomes-expressing cells was restored among CD8+ T cells in the spleen (Fig. 6G), indicating that, once again, the expression of a high-avidity TCR could partially compensate for the absence of Ndrg3. Last, as natural killer T (NKT) cells are reported to be a major source of IL-4 under homeostatic conditions (*65*), we investigated whether NKT cell populations were altered in Ndrg3-deficient mice. Staining with an αGalCer/CD1d tetramer revealed that type I invariant NKT cells were almost completely absent in the thymus (Fig. 6H) and spleen (fig. S8H) of Ndrg3^TKO^ mice. Therefore, the defective expression of Eomes by CD8+ T cells in Ndrg3^TKO^ mice is likely due to both the absence of IL-4–secreting invariant NKT cells and diminished capacity to respond to IL-4.

## DISCUSSION

Our study has revealed that Ndrg3 conditional knockout mice are unable to establish stable populations of peripheral T cells. Instead, these mice exhibit lymphopenia, characterized by the loss of quiescence among naïve T cells, and increased rates of cell death. In the absence of Ndrg3, T cells appeared to proceed through thymocyte development without obvious phenotypic defects; however, mature CD8SP thymocytes exhibited moderate transcriptional changes, such as a frequency of Eomes expression before exiting the thymus. These defects became clearer in the periphery where most CD8+ T cells fail to establish themselves as mature naïve cells in the periphery, and most cells either die by apoptosis or further differentiate into effector/memory-type cells. Although the conversion of RTEs to mature naïve T cells can occur independently of TCR signaling, interaction with major histocompatibility complex (MHC) nevertheless shapes the composition of the more mature repertoire (*66*), and we could show that the fate of Ndrg3-deficient T cells was strongly influenced by TCR avidity. Increased levels of CD5 expressed by the few surviving cells in the periphery of Ndrg3-deficient mice indicated that these cells could be skewed toward the expression of high-avidity TCRs, suggesting that cells capable of strong tonic signaling may be at least partially resistant to the loss of Ndrg3. Subsequent experiments comparing high- and low-avidity TCR transgenes revealed that expression of a high-avidity TCR allowed for a partial rescue of Ndrg3-deficient naïve T cells in the periphery. Our observations are supported by previous studies reporting that high-avidity OT-I or P14 TCRs can facilitate the survival of naïve T cells (*67*–*69*) under adverse conditions. For example, Kdelr1-mutant mice exhibit reduced populations of naïve T cells because of increased activation of the integrated stress response pathway, and this phenotype can be alleviated by the expression of high-avidity TCRs (*67*, *68*). Whether Ndrg3-phenotype rescue in P14+ naïve T cells is the consequence of reduced integrated stress response activity remains to be investigated.

One perplexing observation from our study was that while Ndrg3-deficient cells lose quiescence and are more proliferative in vivo, they proliferated much less than control cells when intentionally activated in vitro. Therefore, it would seem that Ndrg3 influences homeostasis- and antigen-driven proliferation in different ways. Rather than proliferate, stimulated Ndrg3-deficient cells instead secreted copious amounts of IFN-γ and TNF-α. In this instance, expression of the high-affinity P14 TCR was unable to correct this defect, so although strong tonic signaling could correct homeostatic division rates under nonchallenged conditions, the resulting naïve cells were still abnormal in their capacity to expand after activation. Given that naïve Ndrg3-deficient T cells can be activated, as demonstrated by CD69 up-regulation and cytokine production, we suspect that the defect in activation-induced proliferation is linked to an altered capacity of naïve T cells to differentiate into effector cells. This would fit with the observation that the frequency of Eomes expression is reduced among Ndrg3-deficient cells, while Tbet, which is known to promote the generation of short-lived effectors (*70*), is increased. Modulating naïve and memory cell fates in response to either homeostatic or activatory signals therefore appears to be a key function of Ndrg3.

In keeping with this, we also demonstrated that Ndrg3 plays an important role in facilitating IL-4–induced Eomes expression. A diminished frequency of Eomes expression has significant ramifications for the development of innate or VM cells, which we also show to be reduced in Ndrg3-deficient mice; however, it cannot alone explain the lymphopenic phenotype, as Eomes-deficient mice have largely normal populations of naïve T cells (*71*). IL-4–mediated induction of Eomes expression is highly dependent on Akt, and other Ndrg-family members have previously been shown to modulate PI3K-Akt signaling in T cells. For example, Ndrg2 is a known regulator of PI3K-Akt signaling in T cells, where it is thought to suppress Akt activation by binding to protein phosphatase 2A and promoting PTEN activity (*36*). Furthermore, ndrg1b overexpression has also been shown to suppress overactivation of the PI3k-Akt pathway during thymus development in PTEN-deficient zebrafish (*33*); therefore, mounting evidence suggests that Ndrg-family members are important regulators of signaling pathways in T cells. Given that the TCR signal strength dictates the outcome of downstream signaling pathways, including those mediated by Akt (*72*, *73*), we speculate that Ndrg3 is required for naïve T cells to successfully integrate TCR and cytokine signals, especially in cases when TCR avidity is low.

Previous work has demonstrated that the expression of self-reactive TCRs can drive CD8+ T cells to adopt a memory phenotype, with characteristic high-level expression of Eomes. This phenotype is imprinted in the thymus, where a small fraction of CD8SP first up-regulates Eomes, but it is only after a consolidation phase in the periphery that self-reactive cells destined to adopt a memory phenotype become almost uniformly Eomes+ (*56*). Our data indicate that Eomes up-regulation is compromised in both the induction and consolidation phases in Ndrg3-deficient mice, as the proportion of Eomes-expressing cells is already decreased at the CD8SP stage and never reaches normal proportions in the periphery. Although expression of the high-avidity P14 TCR was unable to correct the defective induction of Eomes by Ndrg3-deficient CD8SP thymocytes, it was nevertheless able to facilitate the consolidation phase of Eomes expression in the periphery, as evident by the restoration of normal proportions of Eomes+ cells in the spleen. Our future work will therefore aim to establish how Ndrg3 modulates such pathways to promote the maintenance of a heterogeneous naïve T cell pool that bears a diverse repertoire of TCRs with varying affinities.

## MATERIALS AND METHODS

### Mice

Ndrg3^tm1a(KOMP)Wtsi^ “knockout-first” mice were generated from embryonic stem (ES) cells obtained from the University of California, Davis, KOMP repository [MMRRC_059209-UCD, clone EPD0025_4_B01 (*74*)]. These mice were then crossed with ACTB:FLPe mice [RRID:IMSR_JAX:003800; (*75*)] to remove the LacZ/neoR reporter cassette, resulting in a conditional *Ndrg3*^*tm1c(KOMP)Wtsi*^ allele in which a critical exon (encoding nucleotides 444 to 549 of the reference transcript NM_001355391.1) of the *Ndrg3* gene is flanked by loxP sites. The pLck-Cre (*39*), Rag2-EGFP (*43*), HY-TCR (*51*), and P14 (*52*) mouse lines have all been previously described and were maintained on a C57BL6/J background. Both male and female mice were used and analyzed at 6 to 9 weeks of age, unless otherwise specified. Mice were bred and housed under conventional conditions at the Max Planck Institute of Immunobiology and Epigenetics animal facility. All animal experiments were performed in accordance with local regulations and were approved by the review committee of the Max Planck Institute of Immunobiology and Epigenetics and the Regierungspräsidium Freiburg, Germany (license Az 35-9185.81/G-12/85).

### Flow cytometry

Lymphocytes were isolated by gently pushing spleens and thymi through 40-μm cell strainers (431750, Corning) to obtain a single-cell suspension in fluorescence-activated cell sorting buffer [phosphate-buffered saline (PBS), 0.5% bovine serum albumin, 0.02% NaN_3_]. Splenic cell pellets were additionally treated with ACK lysis buffer (0.154 M NH_4_Cl, 0.01 M KHCO_3_, and 0.1 mM EDTA) to remove erythrocytes. Surface staining and all wash steps were performed in fluorescence-activated cell sorting buffer. A total of 10^6^ cells was pelleted, washed, and centrifuged. To stain surface antigens, cells were incubated with fluorescently labeled antibodies and F_C_ block (14-0161-85, Thermo Fisher Scientific, 1/1000) for 30 min at 4°C in the dark. The Gp33 tetramer labeled with PE was a gift from H. Pircher, and tetramer staining was carried out at the same time as surface antibody staining. All antibodies and working dilutions are listed in table S1. Cell viability was determined by staining with hydroxystilbamidine dye (1 μg/ml; Fluoro-Gold, ab138870, Abcam)*.* All centrifugation steps were performed at 4°C and 400*g* for 4 min. Intracellular staining was performed using the eBioscience Foxp3/Transcription Factor Staining Buffer Set (00-5523-00, Invitrogen) as per the manufacturer’s recommendation. Apoptotic cells were identified by staining with the FITC Annexin V Apoptosis Detection Kit (640914, BioLegend). Briefly, cell pellets were resuspended with 50 μl of annexin V-FITC (1:20) diluted in Annexin V Binding Buffer and were incubated for 15 min at room temperature (RT) in the dark. After incubation, 200 μl of Annexin V Binding Buffer was added to the well with viability dye (hydroxystilbamidine) at a final dilution of 1/1000. Cell cycle analysis was carried out using intracellular Ki-67 stain followed by incubation with 150 μl of 4′,6-diamidino-2-phenylindole (DAPI; 1 μg/ml). Samples were incubated in the dark for 30 min at RT before analysis. For the detection of active caspase-3, thymocytes were first surface stained, followed by anti-active caspase-3-PE intracellular staining for 30 min at 4°C (*41*). Flow cytometry data were acquired using LSRFortessa instruments (BD Biosciences). Acquired data were analyzed using FlowJo Software (FlowJo version 10.10, BD Biosciences).

### T cell proliferation assay

Naïve T cell proliferation assays were conducted by first labeling the total splenocyte suspension with 5 μM cell proliferation dye (CPD-eFluor450, 65-0842, eBioscience) for 10 min at 37°C in the dark. Labeling was stopped by the addition of 40 ml of cold Iscove’s modified Dulbecco’s medium (IMDM; Gibco) and 10% fetal bovine serum (FBS), and cells were immediately centrifuged (4°C, 400*g*, and 15 min). Labeled cells were washed with 10 ml of IMDM and 10% FBS, centrifuged (4°C, 400*g*, and 8 min) and surface stained for naïve CD8+ T cell markers, and sorted as CD8+CD62L+CD44^low^ cells. After sorting, naïve cells were pelleted, and 8 × 10^4^ cells were plated in 200 μl of IMDM and 10% FBS per well in 96-well round-bottom plates with 2 μl per well of Mouse T-Activator CD3/CD28 Dynabeads (11456D, Thermo Fisher Scientific). To activate naïve CD8 T cells with PMA/ionomycin, cells were prepared and sorted as described above. After sorting, naïve CD8 T cells were plated with the addition of PMA (0.2 ng/ml) and ionomycin (200 ng/ml). To stimulate P14+ cells with the gp33 peptide, Thy1.2 P14-expressing naïve CD8 T cells from Ndrg3^ctrl^ and Ndrg3^TKO^ were stained with proliferation dye and sorted. Cells (10^5^) were then plated with Thy1.2+ total splenocytes at a ratio of 1:1 and stimulated with 10^−6^ M gp33 peptide (gift from H. Pircher). Samples from all proliferation experiments were analyzed after 48 hours of stimulation. Cell counts were determined using CountBright Absolute Counting Beads (C36950, Invitrogen). Cytokines in cell culture supernatants were quantified using the LEGENDplex Mouse Inflammation Panel (740446, BioLegend) according to the manufacturer’s instructions. Data were analyzed using software provided by the manufacturer.

### Thymocyte culture with IL-4

Sorted CD8SP MHC1^high^ thymocytes (2 × 10^5^) were plated in 200 μl of IMDM and 10% FBS per well in 96-well round-bottom plates. IL-4 (214-14, PeproTech) was added to the cultures to a final concentration of 20 ng/ml and incubated for 20 hours at 37°C. After incubation, cells were harvested and stained as described above for analysis by flow cytometry. Where indicated, 5 μM Akt inhibitor VIII (124018, Calbiochem) or 25 nM rapamycin (R8781, Sigma-Aldrich) was added to cells during plating.

### Naïve T cell cultures with IL-7

Sorted naïve CD8+ T cells (10^5^; CD8+CD62L+CD44^low^) were plated in 200 μl of IMDM and 10% FBS per well in 96-well plates. Cells were incubated overnight at 37°C in media alone or with IL-7 (217-17, PeproTech) at a final concentration of 5 ng/ml. After incubation, cells were harvested and stained as described above for analysis by flow cytometry.

### Immunoblotting

Cells were lysed in NP-40 buffer (FNN0021, Invitrogen) supplemented with 1/100 Halt Protease and Phosphatase inhibitor cocktail (78441, Thermo Fisher Scientific) for 30 min on ice. Lysates were subsequently centrifuged (4°C, 12,000*g*, and 10 min), and supernatants were incubated at 96°C for 6 min with Laemmli sample buffer. Proteins were separated by electrophoresis on Mini-Protean TGX Precast Gel 4 to 20% (456-8093, Bio-Rad) in tris-glycine-SDS buffer (25 mM tris-base, 190 mM glycine, and 0.1% SDS) at 80 V for 10 min followed by 200 V for 30 min. Proteins were transferred to the 0.2 μM polyvinylidene difluoride (PVDF) membrane, previously activated with 100% methanol. Wet transfer was carried out in tris-glycine buffer (25 mM tris-base and 190 mM glycine) with 20% methanol. Transfer was done at 100 V for 30 min at 4°C. Next, PVDF membranes were blocked overnight in 5% nonfat dried milk powder (AppliChem) dissolved in PBS with the addition of 0.1% Tween 20 (0.1% PBS-T). Membranes were incubated with primary antibodies for 2 hours at RT and then washed three times with 0.1% PBS-T for 10 min. Secondary antibodies were incubated with PVDF membranes for 1 hour at RT and washed again three times. Membranes were developed in Amersham ECL Prime Western blot detection reagent (RPN2232, Cytiva) for 5 min. Proteins were visualized using a ChemiDoc Touch Imaging System (Bio-Rad). The following primary antibodies and dilutions were used: anti-Ndrg3 [clone EPR9010(B), 1/5000, Abcam, no. ab131266), anti-actin (1/10,000, Sigma-Aldrich, no. A2066), anti-HA tag (clone 2-2.2.14, 1/25,000, Invitrogen, no. 26183), and anti-strep-tag II (1/5000, Abcam, no. ab76949). Anti-rabbit-HRP (1/2000, Dako, no. P0448) and anti-mouse-HRP (1/1500, Dako, no. P0447) were used as secondary antibodies. All antibodies were diluted in 5% milk powder dissolved in 0.1% PBS-T.

### Bulk RNA-seq

RNA extraction was performed using RNeasy Plus Micro Kit (Qiagen). Briefly, 4 × 10^5^ CD8SP TCRb+MHC1^high^ thymocytes from Ndrg3^ctrl^ and Ndrg3^TKO^ mice were sorted into 400 μl of RLT Plus lysis buffer and homogenized by pipetting. Genomic DNA from lysate was removed by loading into gDNA Eliminator spin columns, followed by centrifugation (10,000*g*, 30 s). After the addition of 70% ethanol, the RNA was bound to RNeasy MinElute columns, washed with 80% ethanol, and eluted in 15 μl with ribonuclease-free water. Sample quality measurements and library preparation were carried out by the Deep Sequencing facility at the Max Planck Institute of Immunobiology and Epigenetics (MPI-IE). The RNA-seq libraries were analyzed using the mRNA-seq snakePipes pipeline (version 2.5.3) (*76*). Briefly, reads were trimmed with cutadapt (*77*) and then aligned to the mouse genome (GRCm38.p4, release M9) using STAR mapper (*78*). An extensive quality control was done before and after mapping using a suite of tools and the workflow defaults. The gene-level count matrix was generated using featureCounts (*79*), followed by calculation of the difference in gene expression between sample groups of interest using DESeq2 (*80*). Heatmaps were created with pheatmap (version 1.0.12. 2019) and volcano plot with ggplot2. Heatmap values are based on row-wise *z*-score of counts for the top 25 differentially expressed genes. Significantly up- or down-regulated genes were submitted to Gene Ontology enrichment analysis that was performed using clusterProfiler (*81*). Statistical overrepresentation testing was performed with annotation for subontology “Biological Processes” and “Molecular Function,” with a significance *P* value threshold of 0.05 and Benjamini-Hochberg *P*-adjusted method. Clusters containing genes that were significantly up- and down-regulated were compared for common enriched Gene Ontology terms.

### Statistical analysis

Statistical analysis was carried out with GraphPad Prism (version 10). All datasets were tested for normal distribution with the Shapiro-Wilk normality test. Comparison of two datasets with normally distributed data was carried out with two-tailed unpaired *t* tests. Analysis of populations with significantly different SD (measured by *F* test) was corrected with Welch’s test. In the case of multiple unpaired *t* test analysis, the comparison was additionally corrected using the Holm-Šídák method. Datasets that were not normally distributed were analyzed with a nonparametric Mann-Whitney test.
