# Supplementary material for: Ndrg3 is a critical regulator of peripheral T cell maturation and homeostasis
Source: Sci Adv. 2025 Mar 12;11(11):eads5143. doi: 10.1126/sciadv.ads5143 (PMC11900881; doi:10.1126/sciadv.ads5143)
Supplement: Supplementary file 1 — Figs. S1 to S8 Table S1 Legend for data S1 [file sciadv.ads5143_sm.pdf]

Science Advances
AAAS

Supplementary Materials for

Ndrg3 is a critical regulator of peripheral T cell maturation and homeostasis

Julia A. Komorowska *et al.*

Corresponding author: Jeremy B. Swann, swann@ie-freiburg.mpg.de

Sci. Adv. **11**, eads5143 (2025)
DOI: 10.1126/sciadv.ads5143

The PDF file includes:

Figs. S1 to S8
Table S1
Legend for data S1

Other Supplementary Material for this manuscript includes the following:

Data S1

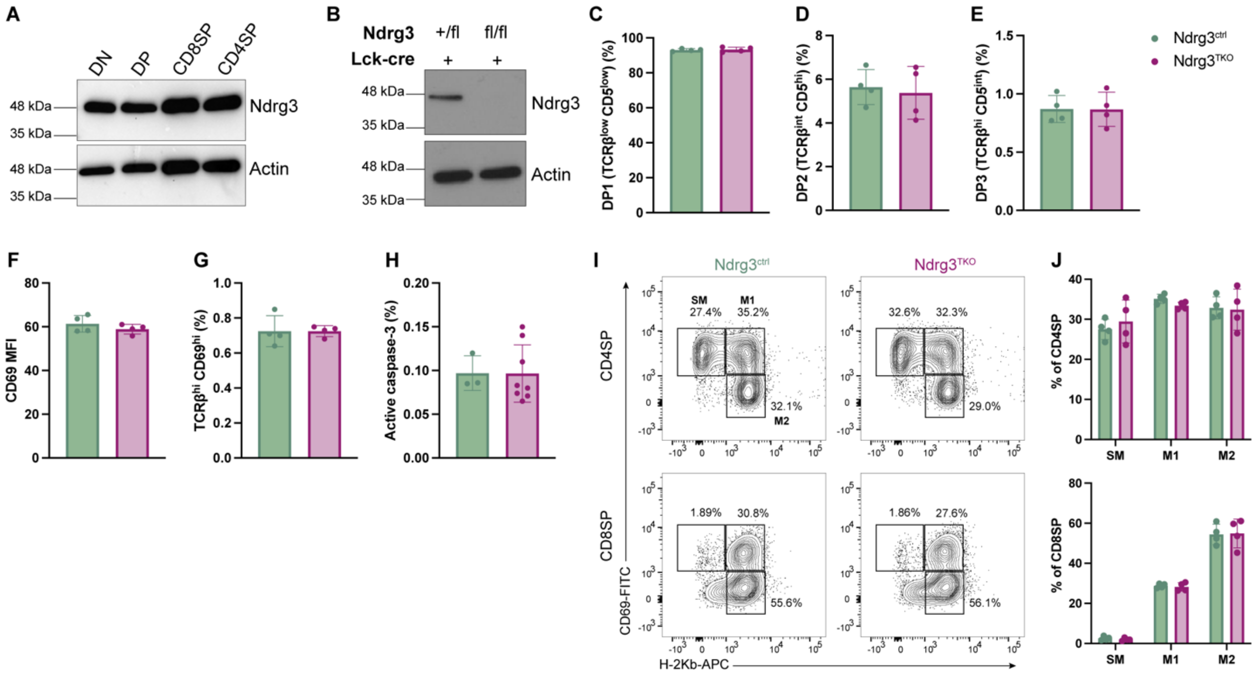

Fig. S1. Analysis of thymocytes from Ndrg3^{ctrl} and Ndrg3^{TKO} mice.

Immunoblots illustrating Ndrg3 expression in sorted DN (excluding CD3+, CD19+, NK1.1+, Gr.1+, Ter119+, MHC-II+ and TCRγδ+ cells), DP, CD8SP and CD4SP thymocyte extracts from wild-type mice **(A)**, and in total thymocyte extracts from Ndrg3^{ctrl} (*Ndrg3$^{+/fl}$;Lck*-cre) and Ndrg3^{TKO} (*Ndrg3$^{fl/fl}$;Lck*-cre) mice **(B)**. Actin was used as a loading control. Maturation stages of DP thymocytes are identified by CD5 and TCRβ markers. DP1 (TCRβ^{low} CD5^{low}), DP2 (TCRβ^{intermediate} CD5^{high}), and DP3 (TCRβ^{high} CD5^{intermediate}) stages are depicted in **(C)**, **(D)** and **(E),** respectively. Expression of CD69 **(F)** and proportion of positively selected TCRβ^{high} CD69^{high} cells **(G)** by DP thymocytes. Proportion of cells undergoing clonal deletion in signaled (TCRβ+ CD5+) total thymocytes with the exclusion of CD25+, NK1.1+ CD19+ and TCRγδ+ cells was measured by expression of active caspase-3 **(H)**. Representative flow cytometry graphs **(I)**and proportions **(J)** of CD4SP (top row) and CD8SP (bottom row) maturation stages defined with CD69 and H-2Kb expression. Percentages indicate CD69+ H-2Kb– semi-mature (SM), CD69+ H-2Kb+ mature 1 (M1), and the most mature (M2) CD69– H-2Kb+ populations. Scatter dot plots are presented as mean ± SD with each symbol representing an individual mouse. Data was collected from ≥2 experiments.

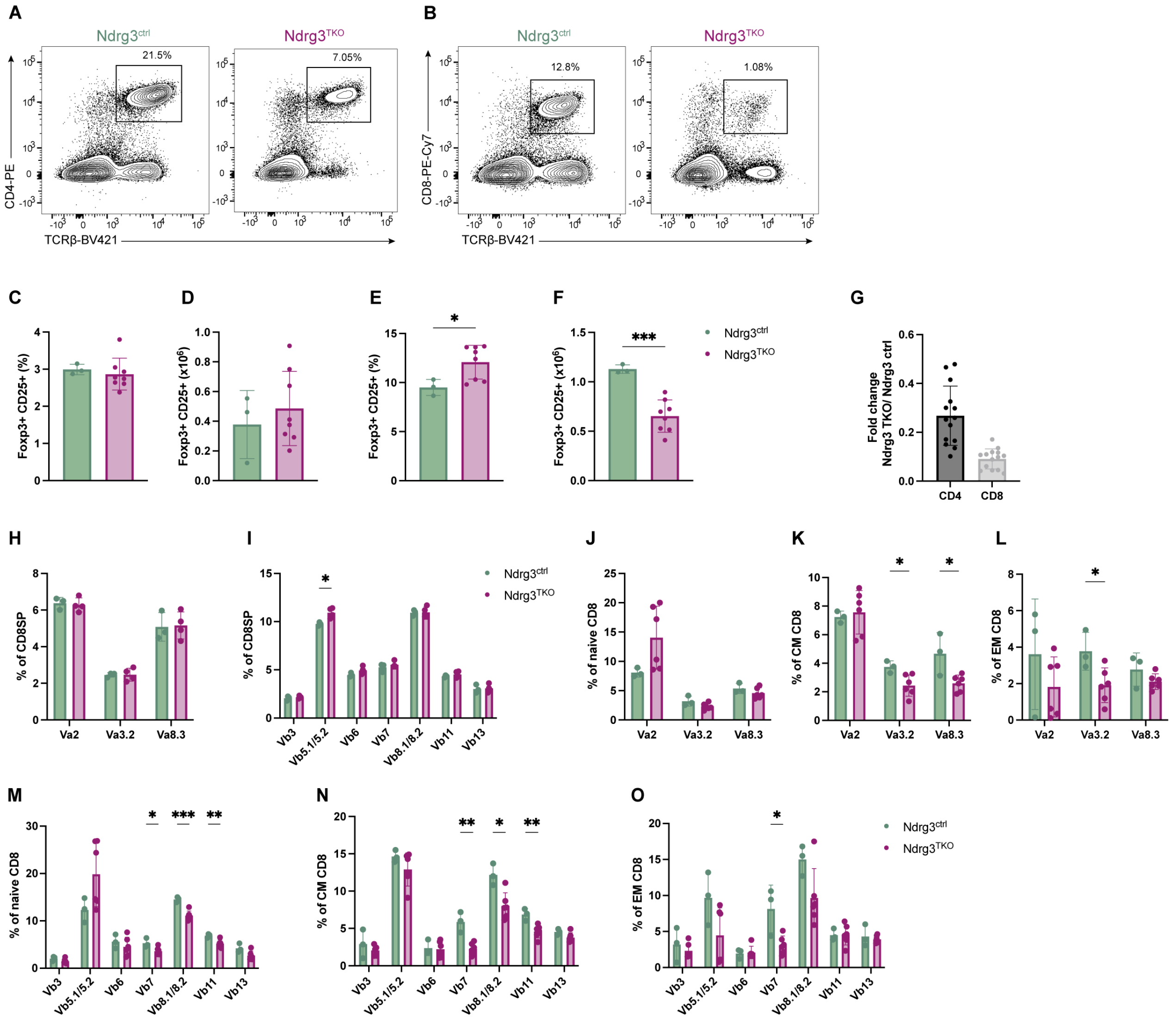

Fig. S2. Characterization of T cell subsets from Ndrg3^{ctrl} and Ndrg3^{TKO} mice.

Representative flow cytometry plots showing gating for TCRβ+ CD4+ **(A)** and TCRβ+ CD8+ **(B)** splenocytes from Ndrg3^{ctrl} and Ndrg3^{TKO} mice. Proportions and cell numbers of Tregs (Foxp3+ CD25+) in CD3+ CD4+ thymocytes **(C-D)** and splenocytes **(E-F)**. Fold change of TCRβ+ CD4+ (CD4) and TCRβ+ CD8+ (CD8) cell numbers between Ndrg3^{TKO} and Ndrg3^{ctrl} in the spleen **(G)**. Flow cytometry analysis of Vα and Vβ TCRs usage on CD8SP thymocytes **(H-I)**, and naïve (CD62L+ CD44^{low}), central memory (CM; CD62L+ CD44^{high}) and effector memory (EM; CD62L- CD44^{high}) peripheral CD8+ T cells **(J-O)**. Scatter dot plots are presented as mean ± SD with each symbol representing an individual mouse. Data was collected from ≥3 experiments. Comparison between groups was calculated using unpaired *t*-tests. *$p<0.05$, **$p<0.01$, ***$p<0.001$.

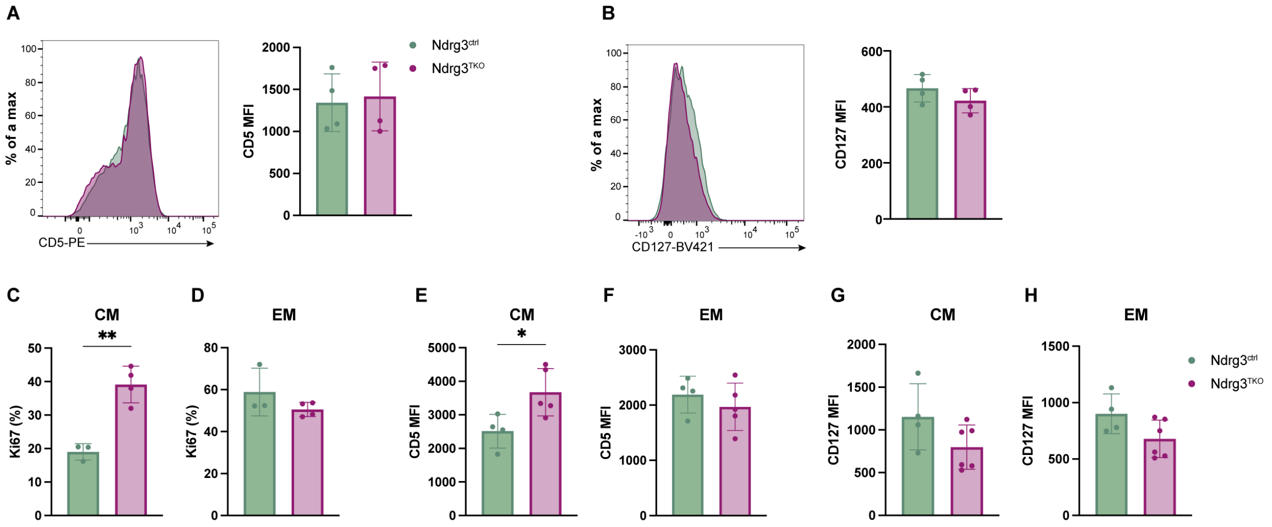

Fig. S3. Detailed characterization of CD8+ T cells from Ndrg3^{ctrl} and Ndrg3^{TKO} mice.

Flow cytometry histograms and median fluorescence intensity (MFI) quantifications of CD5 and CD127 expression in CD8SP thymocytes are shown in **(A)** and **(B)** respectively.

central memory (CM; CD62L+ CD44^{high}) and effector memory (EM; CD62L- CD44^{high}) TCRβ+ CD8+ splenocytes were analysed for the expression of Ki-67 **(C-D)**, CD5 **(E-F)** and CD127 **(G-H)**. Scatter dot plots are presented as mean ± SD with each symbol representing an individual mouse. Data collected from ≥2 experiments. Comparison between groups was calculated using unpaired *t*-tests. *p<0.05, **p<0.01.

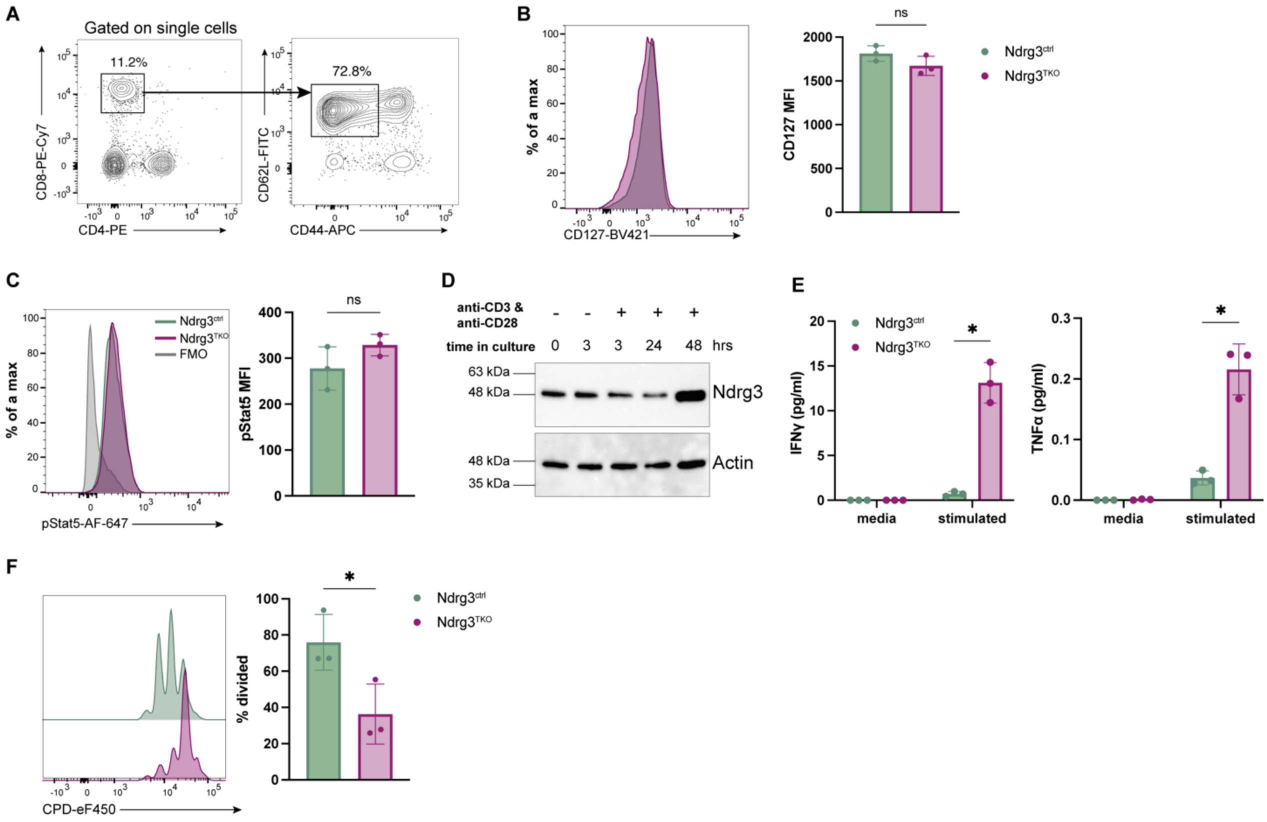

Fig. S4. Ndrg3-deficient CD8+ T cells exhibit impaired response to stimuli.

Representative gating strategy for isolating naïve CD8 T cells by flow cytometry is illustrated in **(A)**. Naïve CD8 (CD8+ CD62L+ $CD44^{low}$) splenocytes were incubated in IMDM supplemented with 10%FCS for 16h at 37°C and probed for the expression of CD127. Histogram and quantification of CD127 MFI are depicted in **(B)**. Naïve CD8 splenocytes were incubated with 5ng/ml IL-7 in IMDM+10%FCS for 16h at 37°C and intracellularly stained for pStat5 (pY694). Histogram and quantification of pStat5 (pY694) are shown are **(C)**. WT naïve CD8 splenocytes were activated for the indicated time with 1ug/ml anti-CD3 and 1µg/ml anti-CD28 at 37°C. Ndrg3 expression was determined in western blot assay as illustrated in **(D)**. Concentrations (pg/ml) of IFNγ and TNFα released to cell culture medium by proliferating (stimulated) or unstimulated (media) naïve CD8 T cells are shown in **(E)**. $8x10^4$ naïve CD8 (CD8+ CD62L+ $CD44^{low}$) T cells from $Ndrg3^{ctrl}$ and $Ndrg3^{TKO}$ mice were labelled with cell proliferation dye (CPD), and stimulated with 0.2ng/ml PMA and 200ng/ml ionomycin for 48h at 37°C. Histograms illustrating proliferation peaks and proportions of divided cells are presented in **(F)**. Scatter dot plots are presented as mean ± SD with each symbol representing an individual mouse. Data collected from ≥2 experiments. Comparison between groups was calculated using unpaired *t*-tests. *p<0.05, non-significant (ns) data indicates p-value of >0.05.

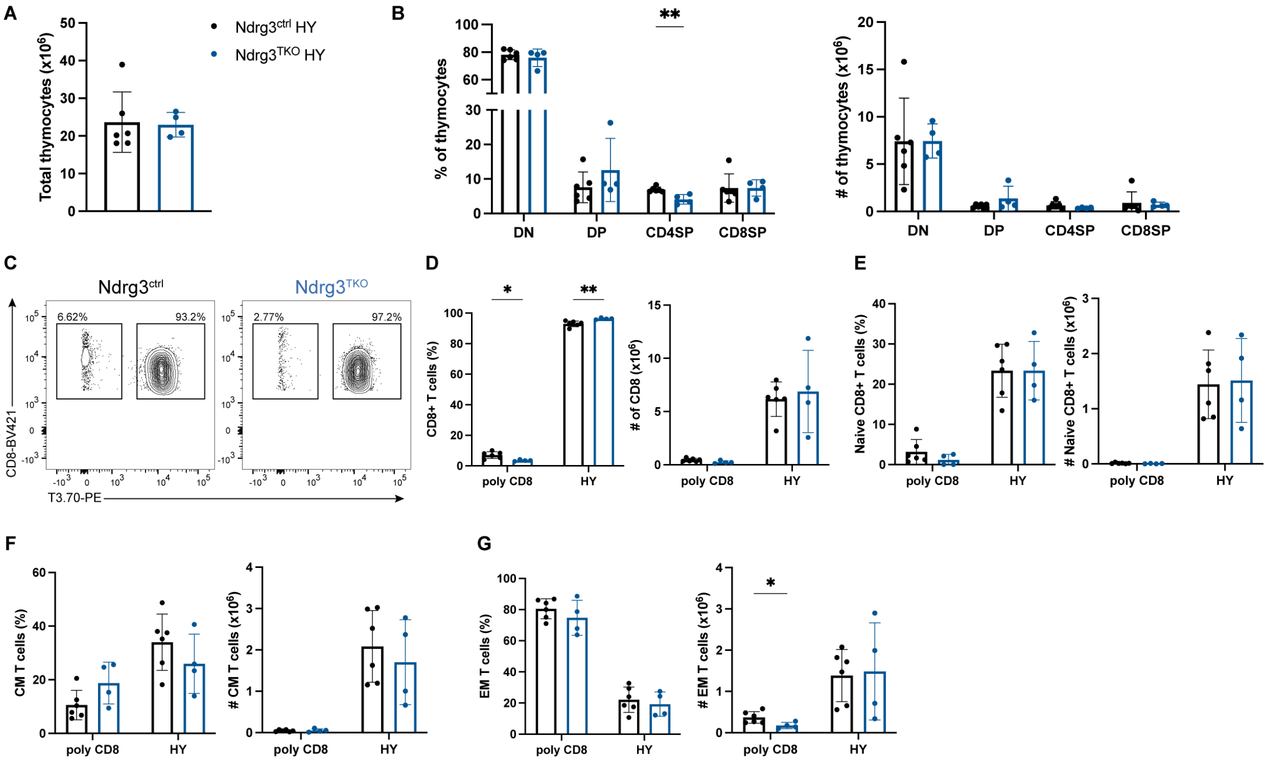

Fig. S5. Proportions and cell numbers of T cells from male Ndrg3^{ctrl} HY and Ndrg3^{TKO} HY mice.

Total thymocyte numbers from 6-week-old male Ndrg3^{ctrl} HY (*Ndrg3$^{+/fl}$;Lck*-cre+;HY) and Ndrg3^{TKO} HY (*Ndrg3$^{fl/fl}$;Lck*-cre+;HY) mice are shown in **(A)**. Proportions and cell numbers of DN, DP, CD4SP and CD8SP thymocytes are depicted in **(B)**. Representative gating of polyclonal CD8 (CD8+ T3.70-) and HY CD8 (CD8+ T3.70+) cells among splenocytes is presented in **(C)**. Proportions and cell numbers of total, naïve (CD62L+ CD44^{low}), central memory (CD62L+ CD44^{high}), and effector memory (CD62L- CD44^{high}) CD8+ T cells are depicted in **(D), (E), (F)** and **(G)** respectively. Scatter dot plots are presented as mean ± SD with each symbol representing an individual mouse. Data was collected from 5 experiments. Comparison between groups was calculated using unpaired *t*-tests. *p<0.05, **p<0.01.

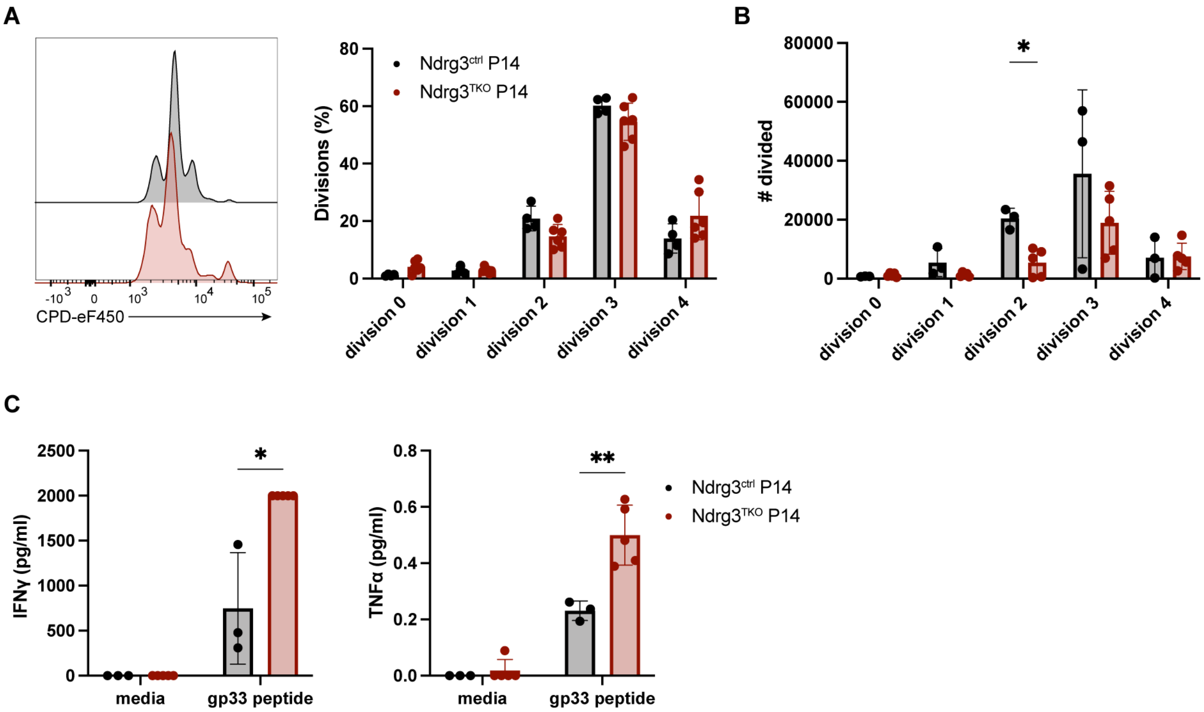

Fig. S6. Proliferative response to gp-33 peptide stimulation by P14-expressing CD8 splenocytes.

Sorted Thy1.2+ naïve CD8 T cells (CD8+ CD62L^{high}) from Ndrg3^{ctrl} P14 (*Ndrg3*$^{+/fl}$*;Lck*-cre+;P14) and Ndrg3^{TKO} P14 (*Ndrg3*$^{fl/f}$*;Lck*-cre+;P14) mice were labeled with cell proliferation dye (CPD) and incubated with Thy1.1+ splenocytes and 10^{-6}M gp-33 peptide for 48h at 37°C. Flow cytometry histograms and quantification of divisions after 48h of stimulation are shown in **(A)**. Cell numbers of proliferating cells in each division peak are depicted in **(B)**. Concentrations (pg/ml) of IFNγ and TNFα released to cell culture medium by proliferating cells are quantified in **(C)**. Scatter dot plots are presented as mean ± SD with each symbol representing an individual mouse. Data was collected from 3 experiments. Comparison between groups was calculated using unpaired *t*-tests. *$p<0.05$, non-significant (ns) data indicates p-value of >0.05.

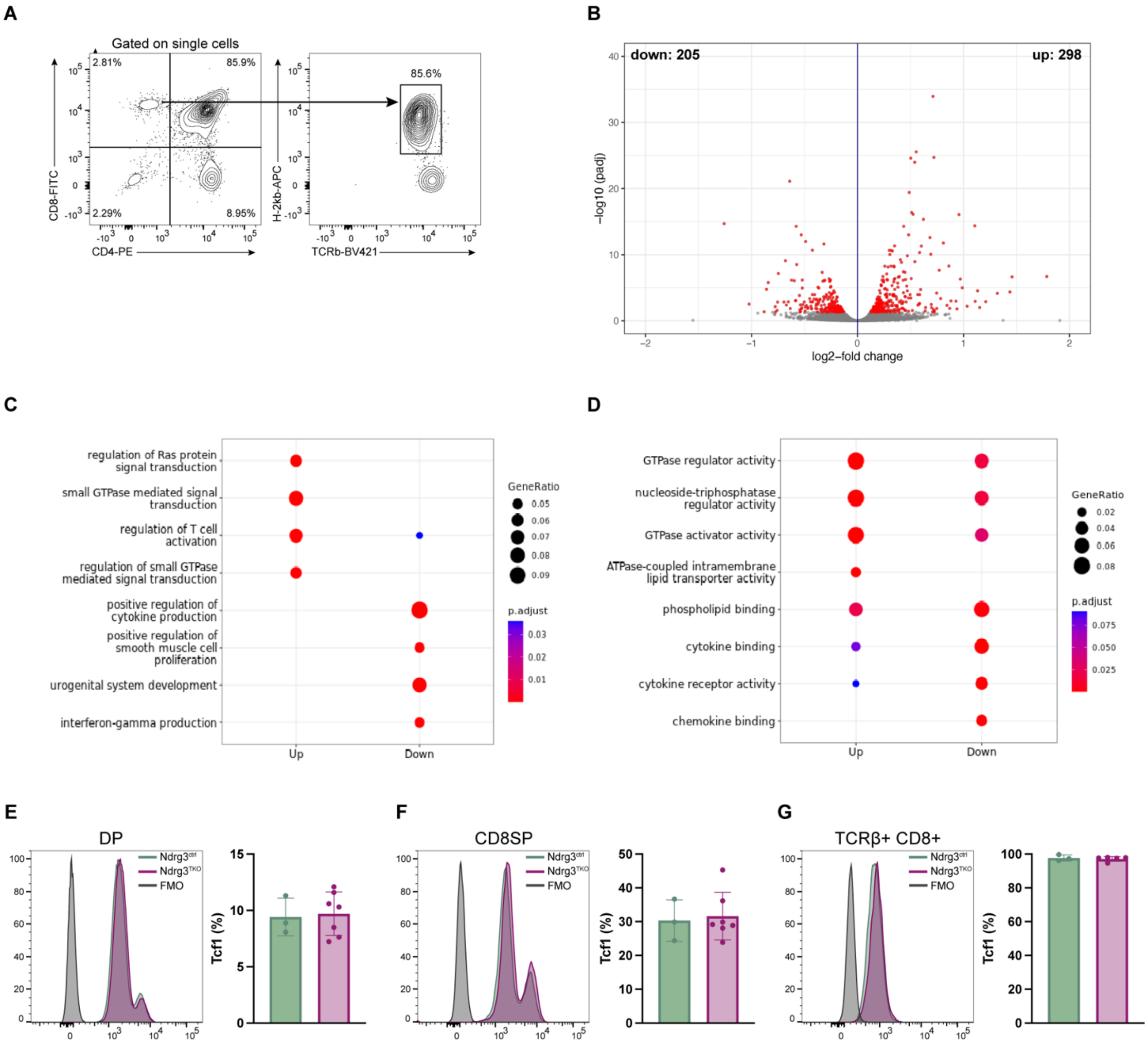

Fig. S7. RNA-seq experiment gating strategy and follow-up analysis.

Gating strategy for isolating CD8SP TCRβ+ MHC-I^{high} thymocytes is shown in **(A)**. CD8SP TCRβ+ MHC-I^{high} cells were sorted and submitted for bulk RNA-seq analysis. Volcano plot showing significantly DEG genes in Ndrg3^{TKO} vs. Ndrg3^{ctrl} CD8SP cells is illustrated in **(B)**. Dots in red represent FDR < 0.05 and LFC > 1. Gene Ontology (GO) overrepresentation of DEG showing biological processes **(C)** and molecular functions **(D)** terms enriched in Ndrg3^{TKO}, padj<0.05 threshold. Histograms illustrate expression and dot plots represent proportions of Tcf1 in DP **(E)**, CD8SP **(F)** and TCRβ+ CD8+ **(G)** T cells. Scatter dot plots are presented as mean ± SD with each symbol representing an individual mouse. Data collected from ≥3 experiments.

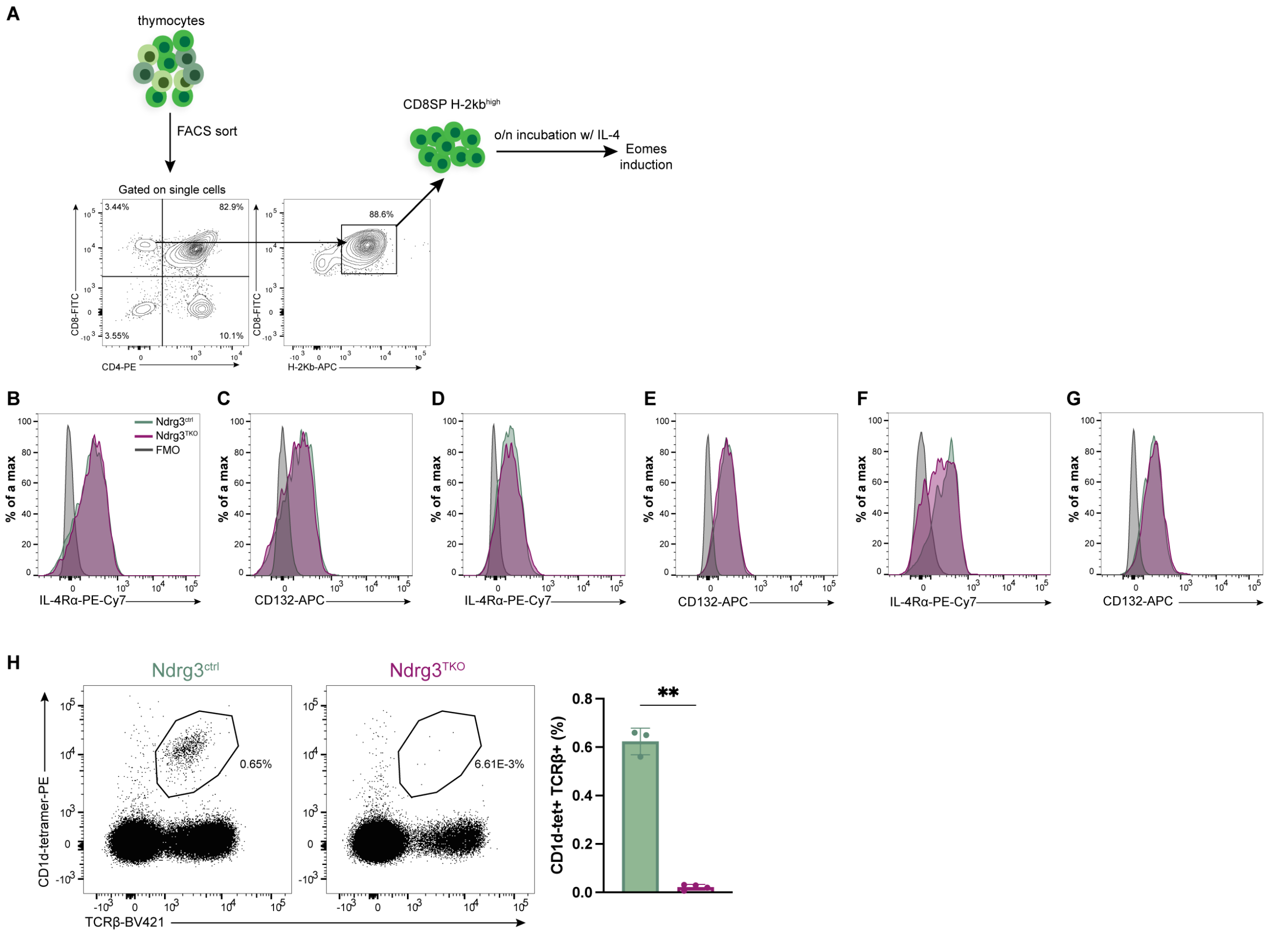

Fig. S8. Characterization of IL-4 receptor components and splenic NKT cells.

Experimental strategy for IL-4 stimulation experiments is depicted in **(A)**. In brief, CD8SP H-2kb^{high} thymocytes were sorted and incubated for 20h with addition of 20ng/ml IL-4, followed by measurement of Eomes induction using flow cytometry. Representative flow cytometry histograms of IL-4Rα and CD132 expression in CD8SP **(B-C)**, TCRβ+ CD8+ CD44^{low} **(D-E)**, and TCRβ+ CD8+ CD44^{high} **(F-G)** splenocytes. Representative gating and quantification of splenic NKT cells (CD1d-tetramer+ TCRβ+) from Ndrg3^{ctrl} Ndrg3^{TKO} mice is shown in **(H)**. Scatter dot plot is presented as mean ± SD with each symbol representing an individual mouse. Comparison between groups was calculated using unpaired *t*-tests. Data collected from ≥2 experiments. **p<0.01.

Table S1. List of antibodies used in flow cytometry experiments.

Antibody	Clone	Dilution	Cat. #	Supplier
Active Caspase-3-PE	C92-605	1/5	561011	BD
CD122-PE	TM-β1	1/100	553362	BD
CD127-BV421	A7R34	1/200	135023	BioLegend
CD132-APC	TUGm2	1/200	132307	BioLegend
CD19-biotin	1D3	1/300	553784	BD
CD1d/αGalCer-tetramer-PE	TC/0147-26agc	1/400	E001-2B-E	ProImmune
CD24-PE	M1-69	1/2000	553262	BD
CD25-APC	PC61	1/500	557192	BD
CD25-biotin	PC61	1/1000	13-0251-85	eBioscience
CD3-FITC	145-2C11	1/500	11-0031-85	eBioscience
CD4-BV421	GK1.5	1/500	100438	BioLegend
CD4-PE	RM4-4	1/2000	12-0043-82	eBioscience
CD4-PE-Cy7	GK1.5	1/1000	100422	BioLegend
CD4-PerCP-Cy5.5	GK1.5	1/1000	100434	BioLegend
CD44-APC	1M7	1/1000	17-0441-81	eBioscience
CD44-PE	1M7	1/400	553134	BD
CD44-PerCP-Cy5.5	IM7	1/400	103031	BioLegend
CD49d-APC	R1-2	1/500	103621	BioLegend
CD5-PE	53-7.3	1/2000	12-0051-82	eBioscience
CD62L-AF-700	MEL-14	1/3000	104426	BioLegend
CD62L-APC	MEL-14	1/1000	17-0621-82	eBioscience
CD62L-FITC	MEL-14	1/1000	11-0621-85	eBioscience
CD69-FITC	H1.2F3	1/200	557392	BD
CD69-PE	H1.2F3	1/400	104508	BioLegend
CD8α-APC	53-6.7	1/800	100712	BioLegend
CD8α-BV421	53-6.7	1/500	100738	BioLegend
CD8α-FITC	53-6.7	1/1000	100706	BioLegend
CD8α-PE-Cy7	53-6.7	1/1000	25-0081-82	eBioscience
CD90.2-APC	53-2.1	1/1000	553007	BD
CD90.2-BV650	30-H12	1/100	740443	BD
EOMES-AF-647	W17001A	1/200	157703	BioLegend
Foxp3-PE-Cy7	FJK-16s	1/100	25-5773-82	eBioscience
H-2kb-APC	AF6-88.5	1/300	116517	BioLegend
IL-4Rα-PE-Cy7	I015F8	1/200	144805	BioLegend
Ki-67-FITC	20Raj1	1/400	11-5699-82	eBioscience
NK1.1-biotin	PK136	1/500	13-5941-85	eBioscience
Tbet-PE	4B10	1/100	644810	BioLegend
TCF1-AF-488	C63D9	1/200	6444	CST
TCR H-Y-PE	T3.70	1/500	12-9930-81	eBioscience
TCR Vα2-APC	B20.1	1/500	127809	BioLegend

TCR Vα3.2-FITC	RR3-16	1/500	135403	BioLegend
TCR Vα8.3-PE	KT50	1/500	125707	BioLegend
TCR Vβ11-PE	RR3-15	1/500	553198	BD
TCR Vβ13-FITC	MR12-13	1/500	553204	BD
TCR Vβ3-PE	KJ25	1/500	553209	BD
TCR Vβ5.1/5.2-APC	MR9-4	1/500	139506	BioLegend
TCR Vβ6-PE	RR4-7	1/500	553194	BD
TCR Vβ7-PE	TR310	1/500	553216	BD
TCR Vβ8.1/8.2-PE	MR5-2	1/500	553186	BD
TCRɣδ-biotin	GL3	1/100	13-5711-82	eBioscience
TCRβ-APC	H57-597	1/800	109212	BioLegend
TCRβ-BV421	H57-597	1/500	109229	BioLegend
TCRβ-PE	H57-597	1/400	12-5961-83	eBioscience
TCRβ-PerCP-Cy5.5	H57-597	1/200	109228	BioLegend

Data S1. Complete list of differentially expressed genes between Ndrg3-deficient and Ndrg3-sufficient CD8SP TCRb+ MHC-I^{high} thymocytes.
